# Exploration of DNA processing features unravels novel properties of ICE conjugation in Gram-positive bacteria

**DOI:** 10.1093/nar/gkac607

**Published:** 2022-07-18

**Authors:** Haifa Laroussi, Yanis Aoudache, Emilie Robert, Virginie Libante, Louise Thiriet, Dominique Mias-Lucquin, Badreddine Douzi, Yvonne Roussel, Isaure Chauvot de Beauchêne, Nicolas Soler, Nathalie Leblond-Bourget

**Affiliations:** Université de Lorraine, INRAE, DynAMic, F-54000, Nancy, France; Université de Lorraine, INRAE, DynAMic, F-54000, Nancy, France; Université de Lorraine, INRAE, DynAMic, F-54000, Nancy, France; Université de Lorraine, INRAE, DynAMic, F-54000, Nancy, France; Université de Lorraine, INRAE, DynAMic, F-54000, Nancy, France; Universite de Lorraine, CNRS, Inria, LORIA, F-54000, Nancy, France; Université de Lorraine, INRAE, DynAMic, F-54000, Nancy, France; Université de Lorraine, INRAE, DynAMic, F-54000, Nancy, France; Universite de Lorraine, CNRS, Inria, LORIA, F-54000, Nancy, France; Université de Lorraine, INRAE, DynAMic, F-54000, Nancy, France; Université de Lorraine, INRAE, DynAMic, F-54000, Nancy, France

## Abstract

Integrative and conjugative elements (ICEs) are important drivers of horizontal gene transfer in prokaryotes. They are responsible for antimicrobial resistance spread, a major current health concern. ICEs are initially processed by relaxases that recognize the binding site of *oriT* sequence and nick at a conserved *nic* site. The ICE*St3*/Tn*916*/ICE*Bs1* superfamily, which is widespread among Firmicutes, encodes uncanonical relaxases belonging to a recently identified family called MOB_T_. This family is related to the rolling circle replication initiators of the *Rep_trans* family. The *nic* site of these MOB_T_ relaxases is conserved but their DNA binding site is still unknown. Here, we identified the *bind* site of RelSt3, the MOB_T_ relaxase from ICE*St3*. Unexpectedly, we found this bind site distantly located from the *nic* site. We revealed that the binding of the RelSt3 N-terminal HTH domain is required for efficient nicking activity. We also deciphered the role of RelSt3 in the initial and final stages of DNA processing during conjugation. Especially, we demonstrated a strand transfer activity, and the formation of covalent DNA-relaxase intermediate for a MOB_T_ relaxase.

## INTRODUCTION

Horizontal gene transfer (HGT) plays an important role in the rapid evolution of bacterial genomes by driving gene exchanges between cells and therefore leading to genetic variability of bacteria. This allows a better adaptation of bacterial populations to their environment and colonization of new ecological niches (1,2). These HGT have also contributed to the emergence of new pathogenic bacteria and to the spread of antibiotic resistance.

Bacterial conjugation is considered to be one of the most important mechanisms of HGT and is defined as the transmission of genetic material from one bacteria to another by direct cell contact (1–3). During conjugation, DNA is transported from a donor to a recipient cell through a conjugation machinery, a type IV secretion system (T4SS) (4). Conjugation is mediated by mobile genetic elements. There are two types of autonomous conjugative elements: conjugative plasmids which are extra-chromosomal elements, and integrative and conjugative elements (ICEs) which are integrated in bacterial chromosomes (5,6).

Genome analyses indicate that ICEs are more widespread than conjugative plasmids in most bacterial phyla, especially in Firmicutes (5,7,8). A major superfamily of ICEs in Gram-positive bacteria is the ICE*St3*/Tn*916*/ICE*Bs1* superfamily (9). Tn*916* and its relatives are involved in the spread of various antimicrobial resistance genes, especially resistance to tetracyclines, macrolides and bacitracin (10–12).These elements were found in a large broad host range among Firmicutes, including severe multi-resistant pathogens such as *Streptococcus pneumoniae*, *Clostridium difficile* or *Staphylococcus aureus* (10,13). Thus, the molecular mechanisms of conjugative transfer are worth to be investigated in this widespread superfamily of ICEs to gain future insights aiming to control its dissemination.

After excision from the bacterial chromosome, ICEs transfer to other cells by using mechanisms similar to those of conjugative plasmids. Thus, ICE conjugation is performed in two distinct steps: (i) processing of the DNA molecule to be transferred by a multi-protein complex called relaxosome, and (ii) translocation of the DNA through a T4SS apparatus from the donor to the recipient cells (4,5). The key enzyme of the relaxosome complex is the relaxase. It initiates conjugation by nicking the DNA at a specific sequence located on the origin of transfer (*oriT*) of the conjugative element, called the *nic* site. This enables its rolling-circle replication (RCR) starting from the 3′OH end of the nicked DNA (14). As relaxases are transesterases, they usually remain covalently attached to the 5′ end of the *nic* site by their catalytic tyrosine (15,16). The nascent DNA-relaxase complex is then recognized and recruited by the coupling protein encoded by the element which directs it to the conjugative T4SS (17). In the recipient cell, the incoming single-stranded DNA is re-circularized by the relaxase (16). The complementary strand is then replicated by host enzymes, and in the case of ICEs, the DNA is then integrated in the chromosome of both recipient and donor strains (5,14).

Conjugative relaxases are classified into different MOB families according to the presence of conserved motifs, phylogenetic analysis and structural data (16,18,19). The ICE*St3*/Tn*916/*ICE*Bs1* superfamily of ICEs encodes non-canonical relaxases that are unrelated to the canonical HUH endonucleases (20). They belong to the MOB_T_ family (7), which is distantly related to the *Rep_trans* RCR initiators encoded by the well-studied pT181 plasmid family (19,21,22). The *nic* site was previously identified for ICE*Bs1* and contains a conserved CT’AA sequence located within the loop of a G/C rich hairpin (23). This *nic* sequence is conserved among ICEs of the ICE*St3*/Tn*916/*ICE*Bs1* superfamily and is identical to the *dso* (double-stranded origin) sequence used by *Rep_trans* proteins to initiate RCR (24,25), strengthening their relationship with MOB_T_ relaxases. Indeed, beyond their role in conjugation initiation, MOB_T_ relaxases encoded by Tn*916* and ICE*Bs1* (Orf20 and NicK, respectively) are also involved in the autonomous RCR initiation of the ICE after excision (26,27).

In contrast to those of conjugative plasmids, only few relaxases from ICEs have been experimentally characterized. So far, three MOB_T_ relaxases have been studied. In 2006, the Orf20 relaxase was surprisingly found to require the cognate Tn*916* integrase for its nicking specificity (28). At that time, 19 putative nicking sites were detected within *oriT*_Tn_*_916_*, without occurrence of the conserved CT’AA *nic* site among them. It is now known that, due to misidentification of the initiator codon, the protein used in the 2006 study was a truncated version of Orf20, deprived from its N-terminal HTH (Helix-Turn-Helix) domain. Therefore, this HTH domain of Orf20 could be required for identification of the *nic* site and for nicking at the correct and unique position, questioning the role of the integrase in this process. Subsequently, this HTH domain was shown to be critical for the RCR initiation activity of Orf20 (27). In 2007, the CT’AA *nic* site of ICE*Bs1* was clearly identified within the loop of a conserved GC-rich hairpin by Lee and Grossman (23), as previously shown for the *dso* of *Rep_trans* proteins. The MOB_T_ NicK relaxase from ICE*Bs1* was involved in this nicking activity, but it was not characterized biochemically. Recently, we characterized another MOB_T_ representative relaxase, RelSt3 encoded by ICE*St3* from *Streptococcus thermophilus* (19,29). RelSt3 is dimeric and harbors a catalytic PF02486 domain which is also present in *Rep_trans* proteins. We demonstrated that RelSt3 active site, as for *Rep_trans* proteins, contains three conserved acidic residues for the coordination of a cationic cofactor (19). However, little is known about how MOB_T_ proteins interact with DNA, and whether a covalent adduct is formed between the cut DNA and a catalytic tyrosine residue of their active site. Several canonical HUH relaxases encoded by conjugative plasmids were studied in some details, revealing that they recognize a *bind* site located close to the *nic* site on the *oriT* sequence. Such examples include the TrwC relaxase (MOB_F_) from *Escherichia coli* R388 plasmid (30), and the TraA relaxase (MOB_Q_) from *Enterococcus faecalis* pIP501 plasmid (31), both of which bind to a sequence located upstream of the *nic* site. This *bind* site usually contains inverted repeats. In other cases, the relaxase is assisted by auxiliary proteins, which can favor the binding and the positioning of the relaxase at *oriT*. For example, the relaxase PcfG (MOB_P_) from the *E. faecalis* plasmid pCF10 is unable to bind DNA directly, but relies on its PcfF partner to do so (32). These auxiliary proteins often harbour a RHH (Ribbon-Helix-Helix) domain responsible for their interaction with DNA (32–35). Instead, *Rep_trans* proteins do not need auxiliary proteins to bind to their *dso* sequence (36,37). Their *bind* site contains an inverted repeat (ICRIII) located immediately downstream of the *nic* site, that allows discrimination of the cognate *Rep_trans* protein (22,37).

Interestingly, most of the MOB_T_ relaxases from ICEs harbour an HTH domain in their N-terminal part. It is thus expected that this HTH domain could bind DNA at *oriT*. In this study, we present how the relaxase RelSt3 interacts with its target binding site in the *oriT* region through its HTH domain. Mapping of the *oriT* sequence required for ICE*St3* conjugative transfer revealed that the RelSt3 *bind* site was unusually distant from the *nic* site. We also reported that the binding of the HTH domain was important for conjugative transfer and for full nicking activity *in vitro*. By using structural modeling, we identified an alpha-helix of the HTH domain involved in the interaction with DNA, which was experimentally confirmed. In addition, we investigated the joining activity of RelSt3 and its covalent binding to the 5′ end of the cleaved DNA. All these data provide significant novel information enabling a better comprehension of how MOB_T_ relaxases are involved in DNA processing during conjugative transfer.

## MATERIALS AND METHODS

### Bacterial strains, cloning and mutagenesis

The bacterial strains and plasmids used in this work are listed in Supplementary Tables S1 and S2. *Streptococcus thermophilus* strains were grown in Lactose (0.5% w/v) M17 broth at 42°C with, when necessary, chloramphenicol (4 μg/ml) and erythromycin (5 μg/ml). In case of mobilization experiments with poriT, spectinomycin was used at 500 μg/ml. *S. thermophilus* LMG18311 harboring ICE*St3* labelled with a chloramphenicol resistance gene was used as a donor strain in conjugation experiments, whereas an isogenic strain without ICE*St3* but harboring the pMG36e plasmid carrying erythromycin resistance was used as recipient cell. *Escherichia coli* EC101 was used for cloning procedures with plasmid pG^+^host9. Deletion of the 5′ part of the *orfJ* relaxase gene encoding the N-terminal HTH domain was done as already described in ref (19), leading to the LMG18311(ICE*St3orfJΔHTHcat*) strain. Plasmid constructs for protein over-expression were built into the *E. coli* DH5α strain and over-expression was performed in *E. coli* BL21(*DE3*).

For mobilization experiments, poriT plasmids containing various fragments of the ICE*St3 oriT* sequence cloned into the pOri1180 plasmid were constructed using *Eco*RI and *Apa*I restriction sites as described in (19). For production of recombinant proteins, the kanamycin resistant pSKB3 vector was used (gift from Stephen K. Burley), introducing a 6 His-tag and a TEV protease cleavage site in frame at the N-terminal end. The pSKB3-RelSt3 vector encoding ICE*St3 orfJ* gene (19) was used as a template for the cloning of RelSt3 sub-domains using *Nde*I and *Hind*III restriction sites. This way, pSKB3 derivatives were generated to produce RelSt3_64–410_ (depleted of its HTH domain) and RelSt3_1–63_ (HTH domain) variants. For the generation of point substitution variants, site-directed mutagenesis was performed by overlap PCR using oligonucleotides described in Supplementary Table S3. For all constructs, the sequence of both strands of the recombinant plasmids was verified by sequencing (Genewiz Inc., Germany).

### Mating experiments

Mating experiments were done as described previously (38). Briefly, *S. thermophilus* LMG18311-derived donor (containing ICE*St3* and harboring a poriT construct for mobilization experiments) and recipient strains were grown overnight in the presence of the appropriate antibiotics. Overnight cultures were diluted to 1:100 and further grown without any antibiotic for the recipient strain, and if appropriate, with spectinomycin (500 μg/ml) for the donor strain to maintain the poriT construct. After growth reached an OD_600 nm_ of 0.4, cells were mixed (1:1) and concentrated 30 times in LM17 broth. For each mating experiment, two 150 μl aliquots of cells were spread separately on 0.45 μm pore-size nitrocellulose filters (Millipore) deposited on soft agar (0.8%) LM17 plates and then incubated overnight at 42°C. Transconjugant (TC) cells were recovered from the filters with 10 mL of LM17, directly spread or concentrated 10 times before plating onto LM17 plates containing chloramphenicol and erythromycin (ICE transfer), or spectinomycin and erythromycin (poriT mobilization). After a 24 h-incubation at 42°C, mating frequency was calculated as the ratio of TC per recipient cell. At least three independent biological replicates were done.

### Protein expression and purification

Over-expression and purification of recombinant RelSt3 (full-length), RelSt3_64–410_, RelSt3_1–63_ and RelSt3 point variant proteins were done as previously described for RelSt3 (19).

### Electrophoretic mobility shift assays (EMSA)

Electrophoretic mobility shift assays (EMSA) were performed in agarose and in acrylamide gels. For agarose EMSAs, 400 ng of each *oriT* DNA produced by PCR were incubated with the indicated amount of RelSt3 protein in a final volume of 20 μl. After incubation for 15 min at 37°C, the mixture was resolved in 1.5% agarose gels in 1× Tris-acetate–EDTA for 1.5 h at 11 V cm^–1^. Gels were then stained with ethidium bromide and images captured with a Bio-Rad XR+ system (Bio-Rad Laboratories, California). Each experiment was reproduced at least three times.

For EMSAs performed with acrylamide gels, DNA substrates were oligonucleotides labelled at their 5′ end with 6-FAM (Eurogentec, France), except for the complementary oligonucleotides that were unlabeled (see Supplementary Table S3 for the list of oligonucleotides). Reactions performed in final volume of 18 μl contained 2 pmol of labelled DNA and increasing amounts of the respective proteins (RelSt3 WT or variants, see figure legends). Reaction buffer contained 20 mM Tris–HCl, 500 mM NaCl and 100 μg/ml of salmon sperm DNA as competitor. The mixtures were incubated for 15 min at 37°C prior addition of 2 μl of loading buffer (2.5 mg/ml bromophenol blue and 0.4 g/ml sucrose). Prior loading samples, 5% (w/v) native polyacrylamide gels (PAGE) were pre-run using miniPROTEAN Bio-Rad cells at 4°C for 1 h at 84 V in 0.5× Tris-Borate-EDTA buffer. Samples were then run at 4°C for 1h 20 min at 84 V. Labelled DNA was visualized using the ChemiDoc XRS system (Bio-Rad) and band intensity was quantified with the Image Lab software (Bio-Rad). At least three replicates were performed for each experiment.

### Hairpin structure of IR2

In order to investigate the putative IR2 hairpin structure, we first submitted the IR2 sequence to the RNAfold web server (39), using DNA parameters. We next performed S1 nuclease assays with the double-stranded ori41 oligonucleotide labelled at the 5′ end of its plus strand with 6-FAM (Eurogentec, France). Reactions were performed in a final volume of 18 μl with 2 pmol of DNA and S1 nuclease at increasing concentrations (see figure legend). Mixtures were incubated for 30 min at 37°C before addition of the loading buffer. The samples were resolved in a 5% (w/v) native PAGE in 0.5× Tris–borate–EDTA buffer for 75 min at 84 V. Labelled DNA was visualized as described for EMSAs.

### Relaxase modeling

The predicted RelSt3 model, as well as the confidence score (pLDDT score) were produced with AlphaFold v2.0 (40) using Colabfold (https://github.com/sokrypton/ColabFold). All 3D models were handled using VMD (41) and VMD-python (https://github.com/Eigenstate/vmd-python). The electrostatic potential was computed using the APBS server (server.poissonboltzmann.org/) (42).

### Construction of a DNA-HTH domain model

The HTH domain from the best scored Alphafold prediction model was extracted and screened against the protein databank (RCSB PDB) with FATCAT 2.0 (43) to search for structural homolog. The resulting hits were further filtered with the RCSB PDB in order to keep only those containing both the HTH domain and the double-stranded DNA (dsDNA). In the best hit structure (PDB ID: 5J2Y), the HTH-like domain was replaced by the Alphafold model of RelSt3 HTH, and optimized by energy minimization with NAMD (41) using CHARMM36 force-field (44).

### Nicking-closing assays

Nicking-closing assays were performed in 18 μl final volume reaction, with the indicated oligonucleotides (2 pmol each) and the protein (RelSt3 WT or variants, see figure legends). We used the same reaction buffer as described for EMSAs to which 5 mM MnCl_2_ was added. Mixtures were incubated for 15 min at 37°C, and treated with proteinase K (1 mg/ml, final concentration) for a second 15 min incubation at 37°C. The resulting oligonucleotides were then run in 5% native PAGE as described for EMSAs. At least three replicates were performed for each experiment.

In order to confirm that the recombinant DNA was generated by religation of ori50 and ori57 oligonucleotides, PCR amplification was performed using a forward primer located on the 5′ end of ori57 oligonucleotide and a reverse primer located on the 3′ end of ori50 oligonucleotide. Amplification was done according to standard procedures provided by the manufacturer using the Phusion High-Fidelity DNA polymerase (Thermoscientific), and the nicking-closing product as template. A 10 sec extension step at 72°C was programmed to obtain a 136-bp PCR product. Electrophoresis of PCR products was performed on 2% agarose gel in 1× Tris–borate–EDTA for 1 h at 110 V. Gels were then stained with ethidium bromide and images captured with a Bio-Rad XR+ system (Bio-Rad Laboratories, California).

### Circular dichroism analysis

Far-UV circular dichroism (CD) spectra were recovered from 190 to 260 nm with a Chirascan Plus spectrophotometer (Applied Photophysics, Ltd, UK) equipped with a Peltier temperature control unit (20°C). RelSt3 WT and variants proteins were dialyzed in CD buffer (50 mM sodium phosphate pH 7.0, 500 mM NaF) and loaded on a flat quartz cell of 0.1 mm path length. Spectra were recorded at a scan speed of 60 nm min^–1^ and 1 nm spectral bandwidth. The average of three scans were converted to mean residue ellipticity (deg cm^2^ dmol^–1^ per residue) using Pro-Dara Viewer software (Applied Photophysics, Ltd, UK).

### DNA-relaxase covalent complex formation

To visualize the formation of DNA-RelSt3 covalent complex, 3′ labelled (6-FAM) ori50 DNA (50 μM) was incubated for 15 min at 37°C with 5 μM RelSt3 (WT or variants) in the reaction buffer containing 5 mM MnCl_2_ (except in the control where 1 mM EDTA was added). When indicated, samples were subsequently treated by nuclease S1 (150 units, ThermoScientific) or proteinase K (1 mg/ml, final concentration). Samples were then resolved in denaturing SDS-PAGE (12%) for 1 h at 180 V in miniPROTEAN Bio-Rad cells. The gel was analyzed using a ChemiDoc XRS system (Bio-Rad) to detect fluorescent labelled DNA and in the case of gel Figure 7A, the same gel was also stained with Coomassie blue to detect proteins.

## RESULTS

### Two distant DNA sequences of the *oriT*_ICE_*_St3_*are required for *in vivo* mobilization by ICE*St3*

**Figure 1.**
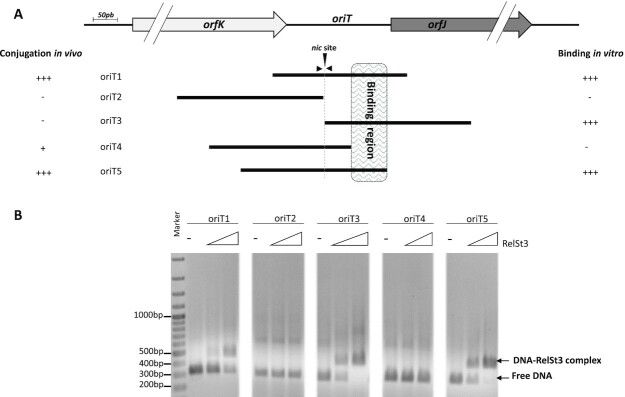
A binding site for RelSt3 distantly located downstream of ICE*St3 nic* site is required for functional conjugation. (**A**) Mapping of the different sequences of the *oriT*_ICE_*_St3_* used for *trans* mobilization assays and EMSA assays, whose results are respectively summarized on the left and on the right. The lines representing each *oriT* segment are to scale. The *nic* site is indicated, flanked by the conserved inverted repeat shown as small arrowheads on the line representing oriT1 sequence. The box with hatched lines materializes the inferred RelSt3 binding site. (**B**) EMSA assays performed with RelSt3 protein on PCR products of each oriT sequence on agarose gel. For each oriT segment, no RelSt3, 0.5 μM and 1.5 μM of RelSt3 were mixed with DNA (400 ng) respectively for the left, middle and right lanes. The ladder is the GeneRuler 100 bp Plus from ThermoFisher.

In a previous study, we showed that RelSt3 functions as a relaxase, displaying a relaxation activity on a supercoiled plasmid harboring the *oriT*_ICE_*_St3_* sequence localized within the *orfJ/orfK* intergenic region of ICE*St3* (19). We also demonstrated that a plasmid harboring this *oriT*_ICE_*_St3_* sequence (corresponding to poriT1 below) was successfully mobilized *in trans* by ICE*St3*. Indeed, this sequence encompasses a conserved *nic* site similar to the one previously identified experimentally for ICE*Bs1* (23). To determine more precisely the nucleotide sequence of the *oriT*_ICE_*_St3_*essential for DNA transfer, 4 other overlapping segments with similar size (≈300 bp) located in the *oriT*_ICE_*_St3_* and adjacent sequences were cloned into the non-mobilizable vector pOri1180, carrying a spectinomycin resistance marker (19). The recombinant plasmids each containing a different segment of *oriT* (oriT1 to oriT5, Figure 1A), were tested for their ability to be mobilized *in trans* by the transfer apparatus of ICE*St3* in *Streptococcus thermophilus* LMG18311 (Table 1). In these assays, conjugation of ICE*St3* was monitored in parallel and was found to occur at similar frequencies (about 1–2 × 10^–3^ TC per recipient cell), regardless the oriT sequence present on the poriT of the donor strain. This indicates that the various *oriT*_ICE_*_St3_* sequences carried by the plasmids did not impair ICE*St3* transfer. As expected, no mobilization of poriT2 and poriT3 plasmids, both lacking the full *nic* site, was observed (below detection level of 10^− 8^). Interestingly, while poriT1 and poriT5 plasmids were successfully mobilized (1–2 × 10^–4^ TC per recipient cell), only very few transconjugants were obtained with poriT4 (Table 1). When normalized to ICE*St*3 transfer, poriT4 mobilization frequency was found to be 50 times lower than that of poriT1. Yet, all three plasmids (poriT1, poriT4 and poriT5) harbor the entire *nic* site. Thus, these results suggested that poriT4 lacks another sequence required for full functional mobilization *in vivo*, which is present in poriT1 and poriT5 and therefore located downstream of the *nic* site. The *oriT*_ICE_*_St3_* sequence present in poriT4 contains 51 bp downstream of the *nic* site, a sequence that is usually long enough to encompass the *bind* site (ICRIII) in the homologous RC replication driven by *Rep_trans* proteins (37). We deduced from these results that the *in vivo* mobilization by ICE*St3* requires an additional sequence, which is surprisingly distant from the *nic* site.

**Table 1.** Mapping of *oriT*_ICE_*_St3_* by plasmid mobilization *in trans*

Plasmid^a^	ICESt3 transfer^b^	Plasmid mobilization^c^	Mobilization ratio^d^
Empty plasmid	1.8 × 10^–4^ ± 1.5 × 10^–4^	<1.0 × 10^–8^	-
poriT1	2.8 × 10^–3^ ± 1.6 × 10^–3^	9.5 × 10^–5^ ± 5.0 × 10^–5^	3.4 × 10^–2^
poriT2	3.9 × 10^–4^ ± 9.9 × 10^–5^	<1.0 × 10^–8^	-
poriT3	1.9 × 10^–3^ ± 2.0 × 10^–3^	<1.0 × 10^–8^	-
poriT4	7.7 × 10^–4^ ± 1.9 × 10^–3^	**4.8 × 10^–^^7^**± 2.5 × 10^–8^	**6.3 × 10^–^^4^**
poriT5	7.8 × 10^–3^ ± 4.2 × 10^–3^	1.9 × 10^–4^ ± 8.2 × 10^–5^	2.5 × 10^–2^

^a^Plasmids harbouring oriT1 to oriT5 sequences as indicated in Figure 1A.

^b^ICE*St3* transfer frequency: number of (EryR-CmR) transconjugants (TC) divided by the number of recipient cells.

^c^Mobilization frequency: number of (EryR-SpecR) transconjugants divided by the number of recipient cells.

^d^Mobilization frequency divided by the ICE transfer frequency.

To determine the location of RelSt3 *bind* site on *oriT*_ICE_*_St3_*, EMSA experiments were conducted on dsDNA substrates corresponding to the five sequences cloned in the poriT plasmids (Figure 1B), produced by PCR amplifications. No shifted band was observed with oriT2 and oriT4 sequences, indicating that RelSt3 does not interact in a stable manner with the sequences located upstream nor immediately adjacent to the *nic* site. In contrast, strongly shifted DNA–protein complexes were observed with oriT1, oriT3 and oriT5 sequences. This localizes the *bind* site for RelSt3 at the 3′-end of the oriT5 segment, within the 78 bp fragment that is absent in oriT4 sequence (hatched box in Figure 1A). Altogether, these results indicated that two sequences of the *oriT*_ICE_*_St3_* are required for DNA transfer: the *nic* site, and a downstream region required for the stable binding of RelSt3. The latter corresponds to an unusually *bind* site distantly located from *nic*, since relaxases from other families usually bind sequences situated in proximity to the *nic* site.

### IR2 within *oriT*_ICE_*_St3_* is the binding site of RelSt3

**Figure 2.**
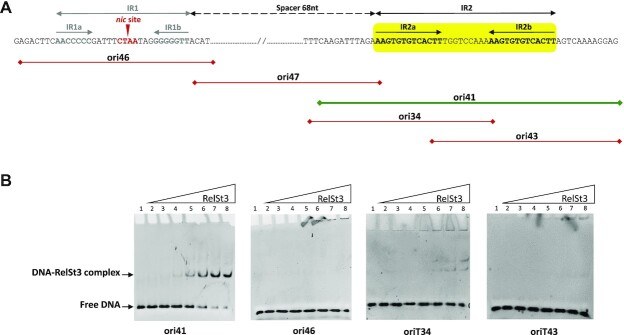
Identification of IR2 as the RelSt3 binding sequence. (**A**) Map of IR and oligonucleotides used in EMSA assays. The position of the IR1 and IR2 are shown on the *oriT*_ICESt3_ DNA sequence. The *nic* site is indicated as a short and red arrowhead within IR1. Solid horizontal lines materialize the dsDNA oligonucleotides used in EMSA experiments, labelled with 6-FAM at the 5′ end. (**B**) EMSA assays illustrating the specific binding of IR2 by RelSt3. The dsDNA-relaxase mixtures were electrophoresed as described in Materials and Methods. Increasing concentrations of RelSt3 were applied as follows: lane 1, 0 μM; lane 2, 0.1 μM; lane 3, 0.2 μM; lane 4, 0.4 μM; lane 5, 0.8 μM; lane 6, 1.6 μM; lane 7, 3.2 μM; lane 8, 6.4 μM. The dsDNA oligonucleotides concentration was 0.11 μM.

Data from the literature indicate that relaxases usually bind to inverted repeat structures located close to the *nic* site. Inspection of the *oriT*_ICE_*_St3_* sequence allowed the detection of two inverted repeats: IR1 and IR2 (Figure 2A). IR1 is a 26-bp sequence, which includes a perfect 7-bp GC-rich inverted repeat (in grey) and an intervening 12 bp containing the conserved CT’AA *nic* site (in red and bold, Figure 2A). IR2 is located 68 bp downstream of IR1 and contains two 13-bp inverted sequences separated by 9 bp. Considering these structures, we divided the *oriT*_ICE_*_St3_* sequence into five regions: ori46 containing IR1, ori41 containing the full IR2, ori47 containing the IR1-IR2 68-bp spacer, and ori34 and ori43 encompassing one of the two parts of IR2 (IR2a and IR2b, respectively). These dsDNA sequences (labelled in 5′ with 6-FAM) were used in EMSA experiments to determine which regions could interact with RelSt3.

As expected, RelSt3 was able to bind the full IR2 (ori41) (Figure 2B) in a dose-dependent manner. Instead, no specific binding was observed with IR1 (ori46) (Figure 2B) nor with the spacer region between IR1 and IR2 (ori47) (data not shown). At high RelSt3:DNA ratio, almost all of the ori41 dsDNA shifted to a DNA–protein complex. The apparent Kd was estimated to be about 1.6 μM (Supplementary Figure S1). No or very poor binding was observed with ori34 and ori43 (Figure 2B) indicating that the whole IR2 is necessary for RelSt3 binding. When EMSA experiments were conducted with single stranded ori41 DNA, or with another non-specific dsDNA of similar size used as a negative control, no binding was observed in both cases, even at high RelSt3 concentrations (Supplementary Figure S2). These data demonstrate that IR2 in a double-stranded form, is the *bind* site for RelSt3 on*oriT*_ICE_*_St3_*.

### Intact IR2 sequence is required for RelSt3 binding

**Figure 3.**
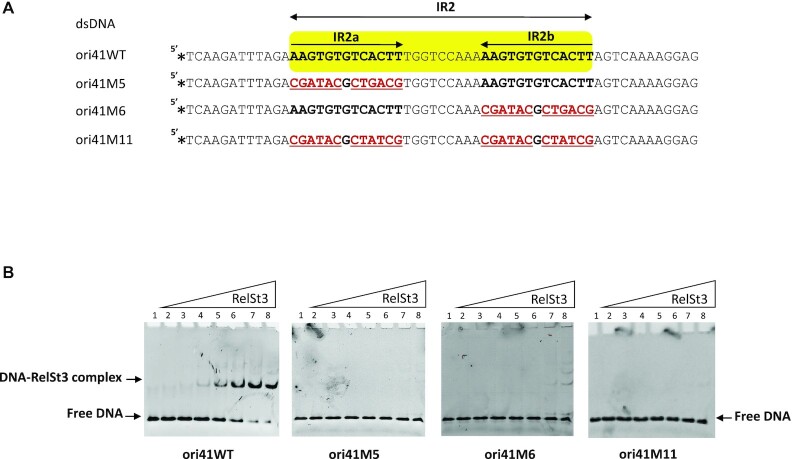
Substitutions in IR2 sequence impair RelSt3 binding. (**A**) Sequence of each substitution mutants used for EMSA assays. IR2a and IR2b are in bold, and substitutions are indicated in bold red and underlined characters. In the upper yellow part, arrows correspond to IR2a and IR2b. Stars indicate the 6-FAM labeling positions. (**B**) EMSA experiments were done as described in Figure 2 with the indicated ds oligonucleotides.

The importance of each individual repeat from IR2 was analyzed by performing EMSA using oligonucleotides harboring mutations modifying one repeat or both (Figure 3A). No binding of RelSt3 was observed when either IR2a (ori41M5) or IR2b (oriM6) sequence was modified (Figure 3B). These results indicated that RelSt3 binding is sequence dependent, and requires both of IR2a and IR2b parts, possibly through the formation of a hairpin structure. The structuration of ori41 ssDNA was investigated by RNAfold using DNA parameters confirming the possible formation of a stem-loop structure at the IR2 (Supplementary Figure S3A) that could result in a cruciform structure of the ori41 dsDNA (Supplementary Figure S3B). To examine further this hypothesis, we incubated ori41 dsDNA with increasing amounts of nuclease S1, an endonuclease that degrades single-stranded DNA. As observed in Supplementary Figure S3C, cleavage products are indeed observed, whose intensity increases with the concentration of S1 nuclease. This result is consistent with the formation of a cruciform structure at IR2.

RelSt3 binding was also tested with a modified sequence harboring an IR of same length and same GC% as IR2 (ori41M11), thus able to form a similar putative hairpin structure. No binding was observed with this ori41M11 substrate, suggesting that RelSt3 binding to IR2 does not only rely on the formation of a hairpin structure but also requires the presence of a specific sequence.

### The HTH domain of RelSt3 is required for *oriT* binding *in vitro* and is essential for conjugation

**Figure 4.**
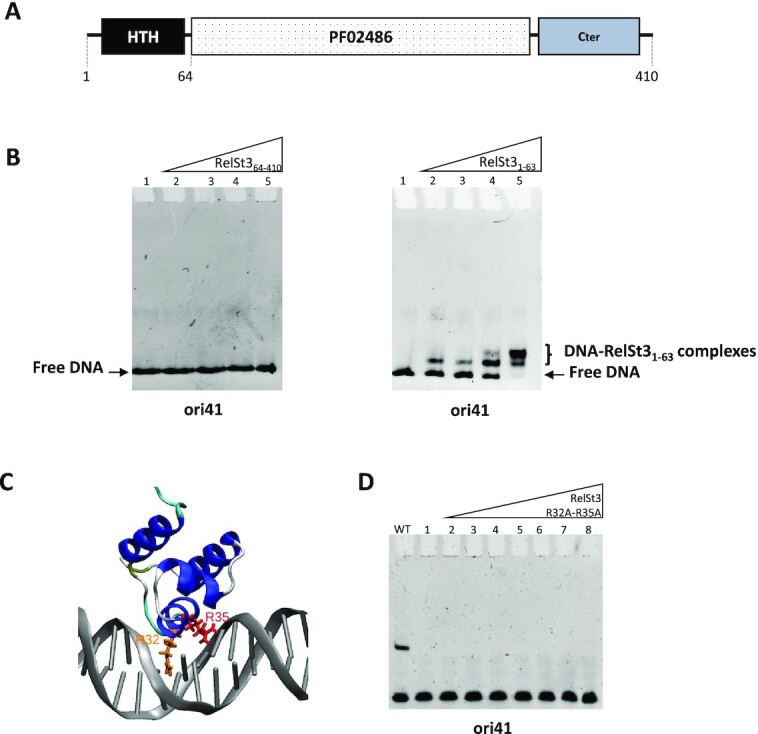
RelSt3 binds to IR2 by its N-terminal HTH domain. (**A**) Organization of the different domains of RelSt3 protein. (**B**) EMSA assays were performed with increasing amounts of RelSt3_64–410_ (left panel) or RelSt3_1–63_ (right panel) incubated with ds ori41 DNA. DNA–protein complexes are observed with the HTH domain of RelSt3 (RelSt3_1–63_) but not with RelSt3 depleted from this N-terminal domain (RelSt3_64–410_). Lane 1, 0 μM; lane 2, 0.5 μM; lane 3, 1 μM; lane 4, 1.25 μM; lane 5, 1.75 μM of protein. (**C**) Model of RelSt3 HTH-DNA complex based on 5J2Y PDB structure. The α-helix extending from residues 30 to 38 of RelSt3 HTH Alphafold model is found in the DNA major groove. The R32 and R35 side chains are shown in yellow and red, respectively. (**D**) EMSA assay performed as described in Figure 2B using the RelSt3 R32A-R35A variant.

At its N-terminal end, from position 8 to 57, RelSt3 displays a predicted Helix-Turn-Helix domain (HTH-XRE, cd00093) that likely functions as a DNA binding domain (Figure 4A). To confirm this hypothesis, we produced a truncated variant of RelSt3 deprived of its HTH domain (RelSt3_64–410_) and used it in EMSA experiments in the presence of ori41 dsDNA. No binding was found even when the RelSt3_64–410_ protein was used at high concentration (Figure 4B). To further assess the role of the HTH domain in *oriT* DNA binding, the variant RelSt3_1–63_, encompassing the HTH domain, was produced and used in EMSA experiments. Even at low concentration of RelSt3_1–63_, DNA–protein complexes were observed indicating the HTH domain of RelSt3 is responsible for its stable binding to *oriT*_ICE_*_St3_*.

To test the importance of this recognition between the HTH domain and DNA in the context of *in vivo* conjugative transfer, a mutant strain of *S. thermophilus* LMG18311 harboring ICE*St3* with a *relSt3* (*orfJ*) gene depleted from its HTH domain was constructed. Mating experiments were carried out using as donor strains, either a *S. thermophilus* LMG18311 strain with the wild-type ICE*St3* (control) or the mutated *orfJΔHTH* strain. While transconjugants were obtained in the control experiment, none were observed using the *orfJΔHTH* strain (with a detection limit <1.0 × 10^–8^ TC/recipient). Altogether, these results demonstrate that the HTH domain of RelSt3 is essential for the conjugative transfer of ICE*St3* since it ensures the binding of RelSt3 to its cognate *oriT* region.

### Modeling of RelSt3 and of the HTH-DNA complex

The structure of the full RelSt3 protein was predicted using Alphafold (40) (Supplementary Figure S4). Five distinct models were obtained, with a highest RMSD between backbones of 5.4 Å. This denotes that these models are close to each other, providing a high degree of confidence. In all these RelSt3 models, the HTH domain was clearly separated from the PF02486 domain by a flexible 10-residues linker. Looking at the electrostatic surface potential of RelSt3 (Supplementary Figure S4), two main positively charged areas were identified: one in the region surrounding the catalytic tyrosine that ensures DNA processing (19), and a second one in the RelSt3 HTH domain that interacts with the *oriT* DNA.

To get further information on the RelSt3 HTH-DNA complex, structural homologs of RelSt3 were recovered from databases (2560 hits, see Materials and Methods) and later filtered to keep only those consisting of protein structures bound to double-stranded DNA (149 hits). The best confidence score (p-value of 2.27 × 10^–08^) was found with the PDB ID 5J2Y, a structure of the DNA-bound RsaL repressor of *Pseudomonas aeruginosa* (45). In the absence of an experimental RelSt3-*oriT* complex structure, we investigated which amino acids of the RelSt3 HTH domain could interact with *oriT*. A model was constructed by replacing the HTH structure of 5J2Y by the HTH model of RelSt3 obtained by Alphafold. The superimposition of the RelSt3 HTH with RsaL HTH domain was carried out by aligning their respective α-helices (Figure 4C). It is important to notice that the DNA structure used in our modeling corresponds to the DNA from the 5J2Y and it is only used as a structural reference to predict the putative amino acids closest to the DNA. The α-helix encompassing residues 37–45 in the 5J2Y structure was found facing the DNA major groove. This region corresponds to the residues 30–38 in the RelSt3 HTH model. Among these amino acids, two arginine residues (R32 and R35) harbor their side-chain towards the major groove of the DNA, and thus could play a role in DNA binding (Figure 4C).

To experimentally assess the potential implication of R32 and R35 in DNA-binding, the variant protein R32A–R35A was produced and tested in EMSAs. Substitution of these residues completely abolished ori41 binding (Figure 4D), indicating that this alpha-helix, and more precisely these arginine residues are important for the recognition of IR2 by the HTH domain.

### Binding to IR2 is required for full RelSt3 nicking activity

**Figure 5.**
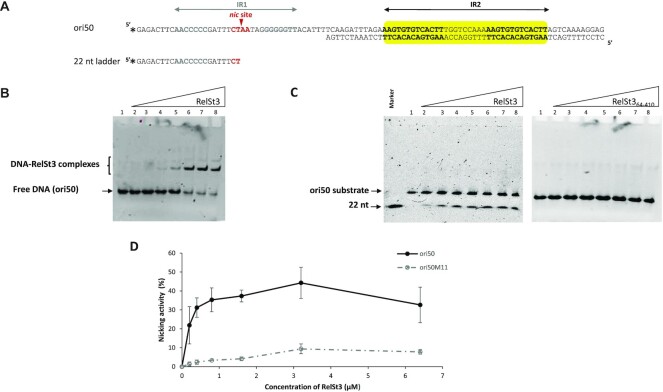
Effective nicking activity of RelSt3 relies on IR2 binding. (**A**) Organization of ori50 substrate. The IR1 *nic* site (single-stranded) and the IR2 binding site (double-stranded) are indicated by arrows above the sequence. The 22 nt single-stranded DNA marker corresponds to the left part of the IR1, generated after RelSt3 cleavage. Stars indicate the 6-FAM labeling positions. (**B**) EMSA experiment performed with ori50 in the conditions described in Materials and Methods. Increasing concentration of RelSt3 were used as in Figure 2. (**C**) Nicking assays were performed in the conditions described in Materials and Methods with the indicated proteins using ori50 as substrate. The same range of increasing concentrations of RelSt3 was used. In the first lane of the RelSt3 gel (on the left), the labeled 22 nt ss-DNA was load alone as marker. (**D**) RelSt3 nicking activity was also assayed with the ori50M11 substrate, and was plotted as a percentage of cleavage of the substrate. Standard deviation of at least 3 replicates are shown.

We previously demonstrated that RelSt3 relaxes supercoiled DNA by introducing a single-stranded nick at the nic site of oriT (19). This nicking reaction requires a divalent metal ion as cofactor such as Mn^2+^. To assess whether RelSt3 binding to IR2 was required for its nicking activity, we designed the chimeric ori50 substrate (Figure 5A). This ori50 DNA substrate contains (i) the *nic* region as a single-stranded DNA to allow easy visualization of the cleavage and (ii) the binding region (IR2) as a double-stranded DNA to ensure the proper fixation of RelSt3 through its HTH domain. We beforehand checked that RelSt3 could bind to ori50 by EMSA experiments (Figure 5B). We found that RelSt3 was able to bind ori50 with an apparent *K*_d_ of 3.2 μM (Supplementary Figure S1), in a similar range to that obtained for ori41 binding.

To examine RelSt3 nicking activity, the wild-type protein was incubated with ori50 in the presence of Mn^2+^. After treatment with proteinase K (thus eliminating DNA–protein complexes), the sample was electrophoresed in native condition. With RelSt3 present at concentrations above 0.4 μM, we obtained a 22 nt DNA, corresponding to the cleavage product at the *nic* site by RelSt3 (Figure 5C). As expected, proportion of this cleavage product increased with concentration of RelSt3. When the RelSt3_64–410_ variant was used in this nicking assay, no cleavage product was observed even at high protein concentrations (Figure 5C). Because this truncated version of RelSt3 lacks the HTH domain, this suggests a direct link between binding and nicking processes. Nicking experiments were also carried out using the ori50M11 as the substrate DNA. Like ori41M11, ori50M11 carries the same modified IR2 (Figure 3A), which was shown to prevent RelSt3 binding. Although a residual nicking activity was observed (Figure 5D), the use of ori50M11 caused a severe decrease of RelSt3 nicking activity. Overall, these results indicate that binding of RelSt3 to IR2 is required for an efficient nicking activity at the *nic* site of *oriT*_ICE_*_St3_*.

### RelSt3 catalyzes strand transfer reaction

**Figure 6.**
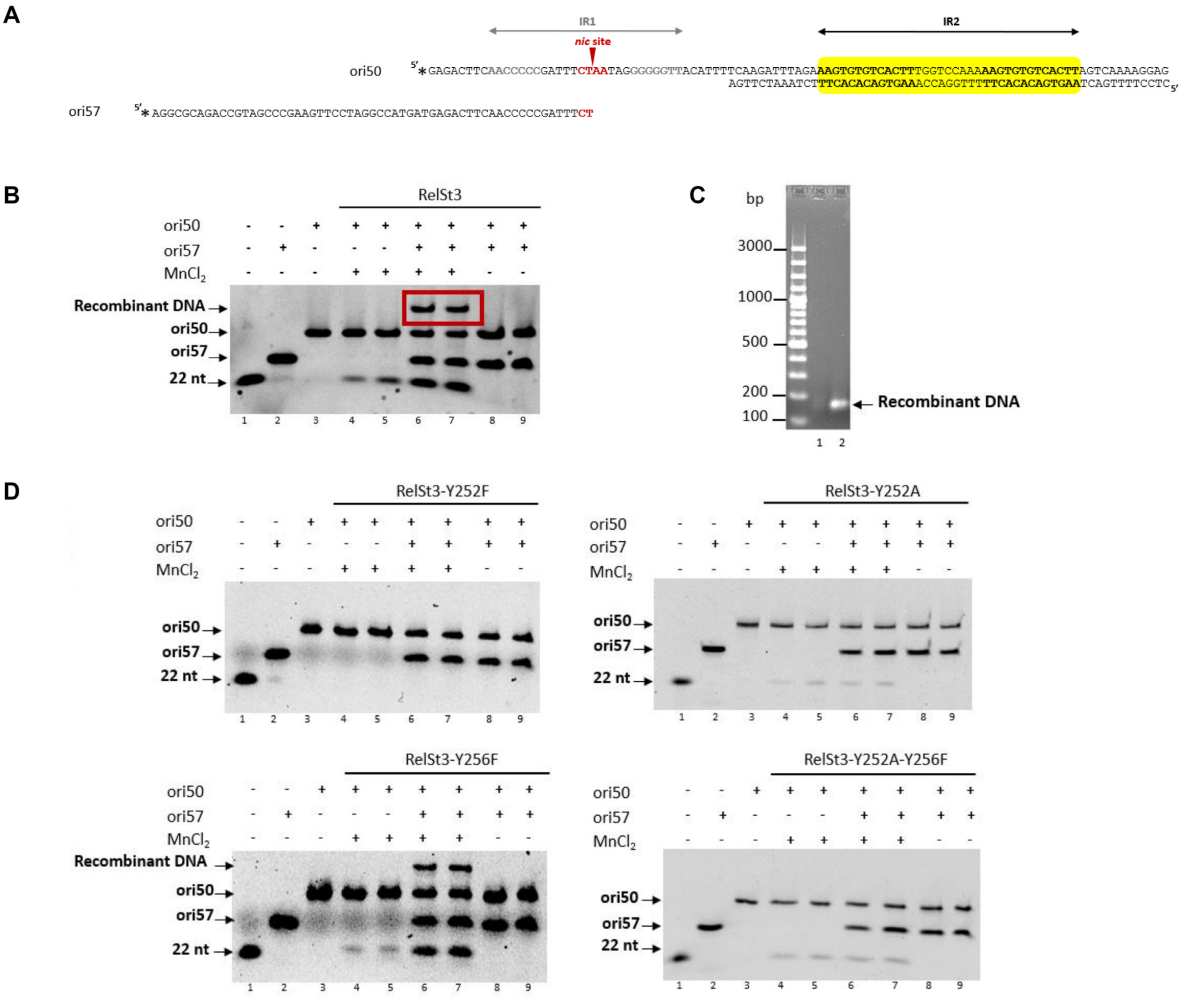
RelSt3 strand transfer activity. (**A**) Position of ori57 with respect to ori50. The organization of ori50 is the same as describe in Figure 5A. Stars indicate the 6-FAM labeling positions. (**B**) Nicking-closing assays performed with RelSt3 WT, in the presence of ori50 and ori57. Lane 1, 22 nt ss-DNA marker; lane 2, 59 nt ss-DNA ori57; lane3, ori50; lanes 4 and 5, ori50 in the presence of RelSt3; lanes 6 and 7, ori50 and ori57 in the presence RelSt3; lanes 8 and 9, ori50 and ori57 in the presence of RelSt3 but in the absence of MnCl_2_ (control). Lanes 4, 6 and 8: 0.8 μM RelSt3. Lanes 5, 7 and 9: 3.2 μM RelSt3. The recombinant DNA obtained by strand transfer is boxed by a red rectangle. (**C**) Electrophoretic migration of the PCR amplifications following strand transfer assays using ori50 and ori53 (lane 1), or ori50 and ori57 (lane 2) oligonucleotides. See Materials and Methods for details. 10 μl of PCR product were loaded on 1% agarose gel. (**D**) Nicking-closing assays performed with RelSt3 active site variants in the presence of ori50 and ori57. The Information concerning the lanes is given in B. The name of the variants is indicated above the vertical lines.

During the conjugation process, relaxases not only initiate DNA transfer in the donor cell but are also thought to recircularize the transferred single-stranded DNA in the recipient cell (4,46). This occurs through a strand transfer reaction mediated by the catalytic tyrosine. In order to explore the putative closing activity of RelSt3, strand transfer reaction experiments were conducted in the presence of two labeled oligonucleotides, ori50 (described in the previous section) and ori57. The ori57 is a 59-mer oligonucleotide corresponding to the sequence upstream of the *nic* position (Figure 6A), so that the 3′ end of this ori57 corresponds to the 22 nt sequence located in 5′-end of ori50 (left hand of *nic* site). Data from these experiments indicate that RelSt3 was able to generate recombinant ori57-ori50 molecules in the presence of Mn^2+^ (red box, lanes 6 and 7, Figure 6B), whereas such product was not observed in the absence of Mn^2+^ cofactor (lanes 8 and 9). We verified by PCR amplification followed by sequencing that it was a genuine recombinant ori57–ori50 molecule (Figure 6C). In the absence of ori57 (lanes 4 and 5, Figure 6B), only the nicking reaction took place, and the initial ori50 substrate should be restored by the strand transfer reaction. This is supported by the fact that the ori50 band remains at a steady intensity with high RelSt3 concentrations. Interestingly, when the strand transfer reaction was done with a truncated version of ori50 (ori56), deprived of the 5′ arm of the *nic* site, no recombinant product was observed (Supplementary Figure S5). This result suggests that a complete *nic* site is needed to be cleaved by RelSt3, this cleavage being the consequence of transesterification by the relaxase active site, which is required to enable strand transfer activity.

### Tyrosine 252 is involved in nicking-closing reactions

Nicking and strand transfer reactions are transesterifications usually involving catalytic tyrosine residues in relaxases active sites (except for MOB_V_ relaxases for which a histidine residue is involved) (16,47). In a previous study, we have assigned the conserved tyrosine 252 (Y252) as the catalytic residue of RelSt3 through plasmid relaxation assays (19). Here, we assessed the involvement of Y252 in the nicking but also in the strand transfer activity of RelSt3 using oligonucleotides. As expected, the replacement of the Y252 by a phenylalanine residue (RelSt3-Y252F) fully abolished the RelSt3 nicking activity, and as a consequence its strand transfer activity (Figure 6D). Unexpectedly, the variant RelSt3-Y252A still showed a low nicking activity, but did not exhibited any strand transfer activity. This result raised the possibility that another catalytic residue could be responsible for the residual nicking activity of the RelSt3-Y252A mutant, and/or possibly participate in the strand transfer activity. In RelSt3, Y252 is enclosed within a conserved motif (motif III) found in both MOB_T_ relaxases and *Rep_trans* proteins. Careful analysis of protein sequence alignments allowed us to identify another conserved tyrosine residue (Y256 for RelSt3) in motif III of MOB_T_ proteins (Supplementary Figure S6, Supplementary Table S4). Even if this residue was not conserved in *Rep_trans* proteins, the motif YxxxY is typical for the Rep proteins of the superfamily I (*Rep_1*) (48,49), whose prototype is the protein A of *E. coli* virus ΦX174 (50). Indeed, viral Reps of this superfamily I use two adjacent tyrosine residues to initiate RCR, alternatively for each replication cycle. In the case of RelSt3, variant Y256F exhibited the same activity profile as the wild type protein (Figure 6BD), indicating that the Y256 is not involved in strand transfer activity. We also used a RelSt3 variant affected on the two tyrosine residues, RelSt3 Y252A-Y256F, to investigate a possible role of Y256 in the low nicking activity observed in the RelSt3 Y252A variant. However, the RelSt3 Y252A-Y256F variant displayed the same nicking-closing activity as the Y252A variant (Figure 6D), indicating that Y256 would not be involved in catalysis. Thus, this residual nicking activity observed with Y252A variant could be due to another residue of the active site, or alternatively, by the nucleophilic attack of a water molecule activated by the metallic cofactor (see Discussion section). This latter hypothesis would also be consistent with the absence of strand transfer activity in RelSt3 Y252A and Y252A-256F variants. We exclude that activity differences found with our variants could be due to major incorrect folding as they displayed similar circular dichroism spectra compared to that of the WT protein (Supplementary Figure S7).

### Trapping the covalent RelSt3-DNA complex

**Figure 7.**
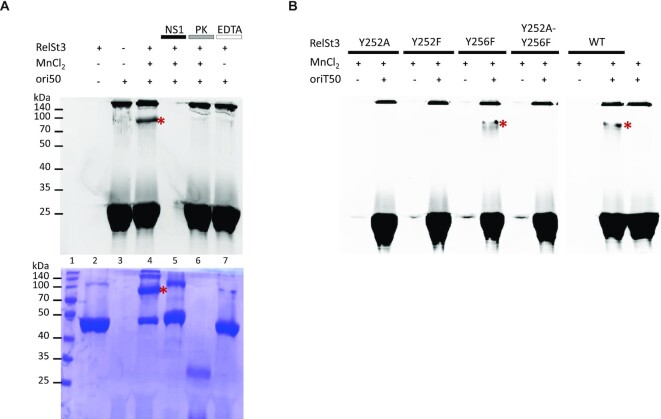
Highlight of RelSt3-DNA covalent complex. (**A**) RelSt3 WT and 3′ labelled ori50 were incubated as described in Materials and Methods in the presence of Mn^2+^. Lane 1: Spectra multicolor broad range protein ladder (ThermoFischer); lane 2: RelSt3 (5 μM, 48 kDa) alone; lane 3: ori50 (50 μM) alone; lanes 4 to 7: RelSt3 and ori50. Control lanes were performed with the addition of EDTA (1 mM) in the absence of Mn^2+^ (lane 7) or with subsequent incubation of nuclease S1 (lane 5) or proteinase K (lane 6). Upper panel: fluorescent imaging. Bottom panel: same gel stained with Coomassie blue. The red star indicates the covalent RelSt3-DNA covalent complex. (**B**) Comparison of covalent complex formation with RelSt3 WT, RelSt3 Y252A, RelSt3 Y252F, RelSt3 Y256F and RelSt3 Y252A-Y256F variants. Red stars indicate covalent RelSt3-DNA covalent complexes.

It is generally assumed that relaxases generate covalent protein-DNA complexes that constitute the substrate for conjugative transfer through T4SS apparatus (4,16). However, in many cases this protein-DNA complex has never been identified experimentally. We thus aimed to capture such a complex formed by RelSt3 and its cognate *oriT*. As the protein is supposed to bind covalently at the 5′ end of the nick, we used an ori50 substrate labelled with 6-FAM at its 3′-end, allowing detection of the protein-DNA complex by fluorescence. Analysis of the gel by fluorescence imaging followed by a Coomassie blue staining would allow both DNA and protein detection to verify the nucleoprotein nature of the complex. As shown in Figure 7, a DNA–RelSt3 complex (red stars in Figure 7A, lane 4) was detected by denaturing SDS-PAGE in the presence of RelSt3 and high concentrations of ori50 (50 μM). The band corresponding to this complex was found to increase with DNA concentration (Supplementary Figure S8). When assays were performed with subsequent incubations with nuclease S1 or with proteinase K, this led to the disappearance of the band corresponding to the nucleoproteic complex (Figure 7A, lanes 5 and 6). This complex appeared only in the presence of the cofactor (Mn^2+^) but not in the presence of EDTA, confirming that its formation relies on the transesterase activity of RelSt3. As expected, when our catalytic tyrosine Y252 variants were used in similar experiments, no complex was detected (Figure 7B). Instead, the relaxase-*oriT* covalent complex was formed with the Y256F variant, where the Y252 tyrosine is intact.

## DISCUSSION

In this work, we have demonstrated that binding of RelSt3 to *oriT* relies on its N-terminal HTH domain, and that RelSt3 binding is a prerequisite for efficient cleavage of the *nic* site *in vitro*. Conjugation experiments also indicated that (i) the binding sequence is required for functional mobilization, and (ii) the HTH domain of RelSt3 is essential for conjugation. These results altogether led us to propose that the binding of RelSt3 through its HTH domain on the *bind* site is an essential prerequisite for subsequent nicking of *oriT* at the *nic* site. As previously observed with other relaxases (30), binding and cleavage would be sequential and distinct steps for the initiation of RelSt3-mediated conjugative transfer. It is reasonable to predict that this sequential process observed for RelSt3 would also be embraced by most of the MOB_T_ relaxases, those harbouring an N-terminal HTH domain (19,27).

Our EMSA experiments showed that RelSt3 was not able to stably bind neither to its *nic* site nor to closely adjacent sequences. We demonstrated instead that RelSt3 only bound to its *bind* site located 68 nt further downstream of its *nic* site. Noteworthy, when we compared ICE*St3 oriT* sequence with those of other related ICEs, we found similar IR2 sequences located at a similar distance downstream from *nic* (Supplementary Figure S9). A minimal consensus sequence ‘TGTCAC’ is almost systematically present, and this may correspond to the basic recognition sequence of the HTH domain of these MOB_T_ relaxases. Interestingly, such IR2 sequence was also detected in the *oriT* of Tn*916*, which is more distantly related (Supplementary Figure S9). This fairly large distance between *nic* and *bind* sites of MOB_T_ relaxases was unexpected. Indeed, many relaxases bind to a sequence close to their *nic* target, directly or through the use of auxiliary proteins. For example, the MobM (MOB_V_ family) and TraA (MOB_Q_ family) relaxases, encoded by the pMV158 and pIP501 plasmids respectively, both bind to an IR adjacent to their respective *nic* site (31,51). However, in other cases, the *oriT* binding step relies on relaxosome auxiliary proteins. In the F plasmid model, even if the mapped binding site of the TraI relaxase (MOB_F_ family) is adjacent to the *nic* site, TraI is thought to be recruited through its auxiliary proteins (52). Especially, the primary binding site of the auxiliary protein TraY (*sbyA*) is located 60–90 bp upstream of the *nic* site, and the major binding sites of TraM (*sbmA* and *sbmB*) are located 200–250 bp upstream of the *nic* site (46). Taking the relaxosome as a whole and considering other systems using auxiliary proteins, the distance between the binding and *nic* sites of *oriT* would not be such an exception.

**Figure 8.**
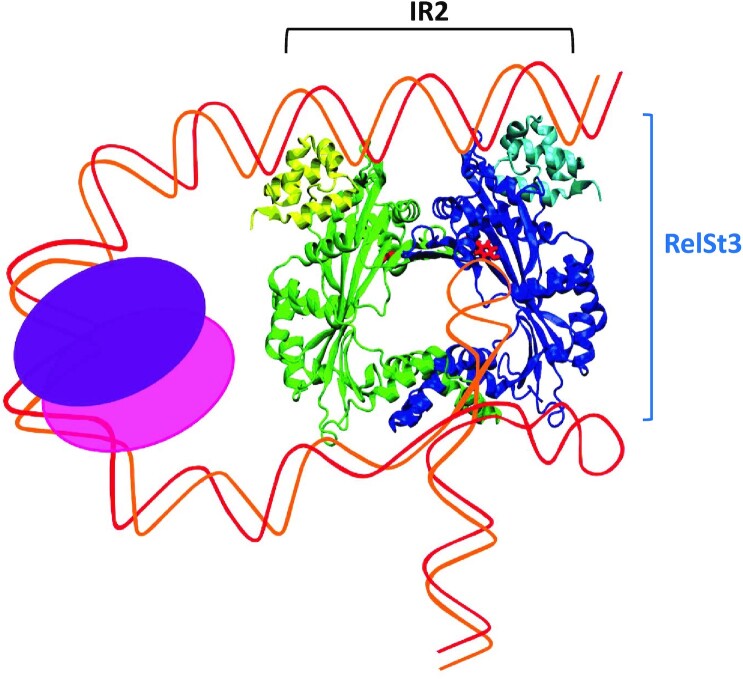
Proposed schematic model for RelSt3 interaction with its *oriT* DNA substrate. The RelSt3 model was obtained using Alphafold. DNA strands are materialized with red and orange lines. The first contact of the relaxase with *oriT* would be mediated by the HTH domain (upper part of the dimer, yellow and light blue), which binds IR2 (*bind* site) with high affinity. Then putative relaxosome partners could bind the DNA in the spacer region (pink and purple), and help bending it in such a way that the induced DNA twists result in IR1 hairpin protrusion inside the relaxase active site. Thus, the active tyrosine of the catalytic domain of RelSt3 (red) would nick the *oriT* at the conserved *nic* site.

Some relaxosome auxiliary proteins have been shown to bend DNA, thus enhancing the nicking activity of the dedicated relaxase. This is the case of TraY, TraM and the cellular IHF protein, which thus facilitate the nicking activity of TraI (53–57). Several other auxiliary proteins were also shown to bend DNA, including for examples the NikA protein from the R64 plasmid (58) or the PcfF protein from pFC10 of *Enterococcus faecalis* (32). Most of these proteins display an RHH DNA binding domain, and their action is important for the recruitment and/or the positioning of the respective relaxase active site towards the *nic* site of *oriT*. In the case of HTH-harbouring MOB_T_ relaxases, the HTH domain likely plays a similar role by binding and probably bending the DNA at the IR2 *bind* site. Indeed, several HTH domains have been shown to bend DNA (59,60). To better understand the interplay between RelSt3 and *oriT* DNA, a 3D model of RelSt3 using Alphafold was obtained. This model indicates that the HTH domain could be separated from the catalytic domain by a flexible linker, and would be located at one extremity of the crescent shape of the catalytic domain. Using the best structural homolog of the RelSt3 HTH domain from the PDB, we built-up a DNA-HTH model leading to the identification of an α-helix probably interacting with the DNA major groove. Suppression of DNA binding using a modified RelSt3 variant (R32A-R35A) affected in this alpha helix confirmed the involvement of these two positively charged arginine residues in DNA binding. Interestingly, electrostatic analysis of the surface of our RelSt3 model highlighted two distinct positively charged regions, which correspond to the two major DNA-interacting regions, the HTH domain and the catalytic site. The absence of other positively charged surfaces on the model suggests that DNA is not bound by the relaxase between the *bind* and the *nic* site. Given the distance between the IR2 *bind* site and the IR1 *nic* site of ICE*St3*, we propose that a fairly large loop may be formed between the IR1-HTH and IR2-active site interfaces. This loop corresponds to the so called ‘spacer’ DNA segment of 68 bp in *oriT* (see our proposed model in Figure 8). We do not exclude that other relaxosome partners could be recruited to bend this DNA region to assist in correct positioning of the IR1 hairpin loop (the CT’AA *nic* site) into the relaxase active site *in vivo*, although they were not strictly required for nicking-closing activities *in vitro*. These putative partners remain to be identified, and could be encoded by the ICE and/or by the bacterial genome.

In turn, the *Rep_trans* RCR initiators from the pT181 family are known to recognize their IRIII *bind* site located next to their IRII *nic* site, via a short string of residues in the C-terminal part of the protein (61,62). This Rep-*bind* site recognition was shown to be responsible for the specificity between the Rep protein and the cognate origin of replication (37). In a seminal study, Carr *et al* solved the 3D crystallographic structure of three *Rep_trans* proteins. Notably, in the RepDE and RepDN structures, it was found that this C-terminal DNA-binding domain was formed by four α-helices arranged similarly to HTH domains (63). These proteins are also dimeric, and surprisingly, the symmetry axis of the DNA-binding domain is tilted 128° with respect to the symmetry axis of the PF02486 catalytic domain. This tilt led the authors to propose a RepDE model interacting with its DNA substrate considering the proximity of the *bind* and the *nic* sites in the *dso*. This is quite different from our presented data. As previously indicated, this DNA-binding domain is located at the C-terminal part of the *Rep_trans* proteins whereas the HTH domain is located at the N-terminal part of the MOB_T_ relaxases. Taken together, all these data are indicative of two different evolutionary pathways leading to the DNA binding function of these transesterases.

Unlike RelSt3, some MOB_T_ relaxases lack the HTH domain, such as the NicK relaxase from ICE*Bs1* (64). As no obvious DNA binding domain has been identified in the genetic context of *nick* gene on ICE*Bs1* genome (65), further studies are needed to understand how these proteins recognize *oriT* DNA. Interestingly, the *Rep_trans* protein RepSTK1 from *Geobacillus stearothermophilus* harbour a C-terminal extended loop in place of the DNA-binding domain of the staphylococcal proteins of the pT181 family (22). The DNA binding interface of RepSTK1 has never been investigated so far, however, this loop could indeed functionally replace the HTH-like DNA-binding domain of its counterparts as it is predicted to be located at a similar position in the 3D structure, and to be positively charged. It is therefore tempting to speculate that MOB_T_ relaxases lacking a HTH domain could bind DNA using a similar loop located in the C-terminal part of these relaxases. With the hypothesis of the presence of such a loop in the 3D structure of NicK in mind, we used Alphafold to obtain a model of NicK (Supplementary Figure S10). Interestingly, a positively charged loop is indeed found for this model, in the same location of the loop found in the RepStK1 structure and the HTH-like DNA-binding domain of RepDE. Even though this loop is much smaller, this result provides clues to the putative DNA-binding domain of MOB_T_ relaxases lacking an HTH domain like NicK, but this remains to be verified experimentally.

When we analysed which catalytic tyrosine residue of RelSt3 were important for nicking-closing activities, a residual nicking activity was unexpectedly found with the Y252A variant. This raised the hypothesis of another intervening residue. As the conserved motif YxxxY had already been described in the active site of some transesterases using two active tyrosine residues (Rep SFI) (48–50), we tested the putative role of the Y256 residue. This was reinforced by the fact that the YxxxY motif was found to be conserved among MOB_T_ proteins. However, the Y256F variant was not altered in its nicking-closing activities, ruling out a catalytic role of Y256 residue in these reactions. This was confirmed with the analysis of the Y252A-Y256F variant, which exhibited the same residual activity observed for the Y252A variant. The residual nicking activity found with the Y252A variant could either be explained by the involvement of another residue in this process, or alternatively, by the action of a water molecule. Indeed, it is possible that in the Y252A variant, the slightly modified folding of the active site would allow the positioning of a metal-activated water molecule in front of the *nic* site. This water molecule could trigger nucleophilic attack when the *nic* site is correctly positioned in the active site. Such metal induced activation of a water molecule has already been described for several types of enzymes, including nucleases (66–69). In line with this hypothesis, we observed no formation of covalent protein-DNA adduct nor religation activity with the Y252A variant. Indeed, such religation can only occur in the case of energy conservation of the ester bond, which is not the case if the nick is catalysed by a water molecule. Taken together, these data argue for a single catalytic tyrosine in MOB_T_ proteins, as previously observed for *Rep_trans* proteins of the pT181 family (22,24).

Our biochemical data demonstrated that the conserved catalytic tyrosine Y252 of RelSt3 was essential for its nicking-closing activities, and for the formation of a stable protein-DNA covalent adduct. These characteristics are shown here for the first time for a MOB_T_ relaxase, and they are likely to be generalizable to other relaxases of the same family. Given that conjugative transfer requires the formation of a stable covalent ssDNA-relaxase adduct, the religation activity described here could be an obstacle to the conjugation process, favouring instead the RCR in the donor cell (26,27). Thus, as yet unidentified regulatory factors may be involved in maintaining the covalent relaxase-DNA complex to promote its recruitment to the T4SS apparatus. Such factors could take part, even transiently, in formation of the relaxosome complex.

During the 90′s, a peculiar regulation mechanism for DNA replication was uncovered for RepC encoded by the pT181 staphylococcal plasmid. The initially active RepC was shown to be converted into a new form after a round of RCR, the inactive RepC* (61,70,71). The latter is still covalently bound through its active tyrosine to a 10–12 oligonucleotide corresponding to the sequence immediately downstream of the *nic* site. Thus, each dimer of RepC can only perform one cycle of pT181 replication, participating in the copy number regulation. Such regulation has never been observed for conjugative relaxases. Given that *Rep_trans* and MOB_T_ proteins share conserved protein sequence motifs and similar 3D folding and as they target identical sequence at *nic* sites (19,22,72,73), a similar regulation of ICE DNA copy number could also be reasonably postulated for MOB_T_ relaxases. Furthermore, as they have already been implicated in autonomous RCR (26,27), several circular copies of ICE could accumulate in the cell, and thus this copy number has to be controlled in some way.

## DATA AVAILABILITY

Source data as well as plasmids and strains are available upon request.

## Supplementary Material

gkac607_Supplemental_Files
