# Supplementary material for: Exploration of DNA processing features unravels novel properties of ICE conjugation in Gram-positive bacteria
Source: Nucleic Acids Res. 2022 Jul 18;50(14):8127–42. doi: 10.1093/nar/gkac607 (PMC9371924; doi:10.1093/nar/gkac607)

Supplementary Figure S1

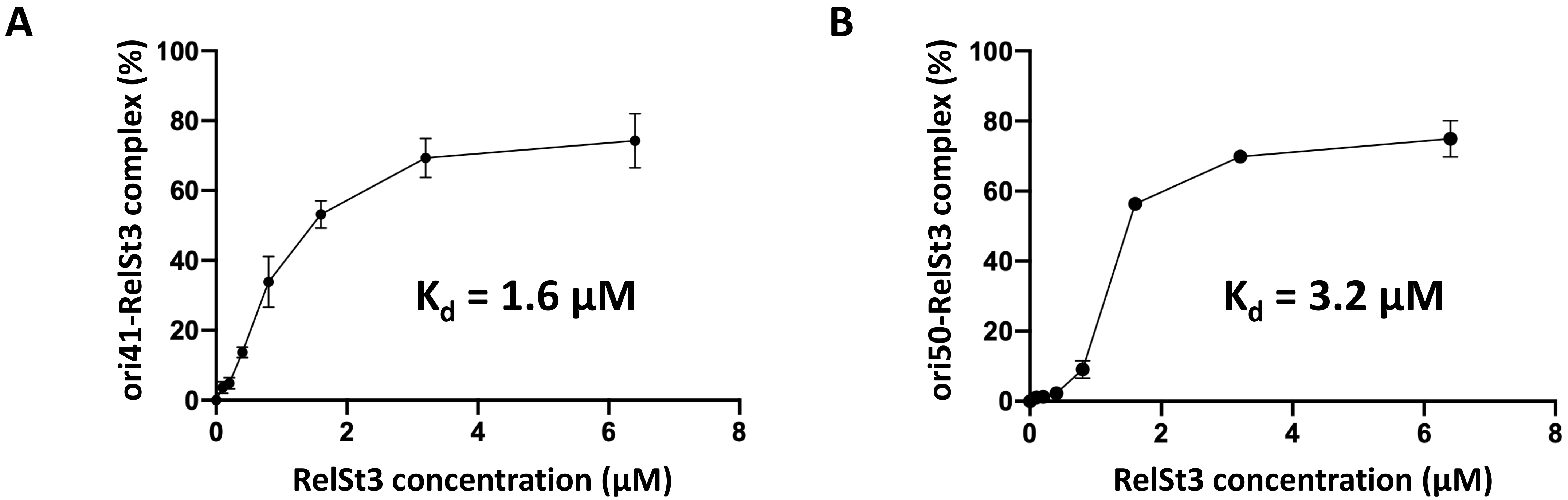

Supplementary Figure S2

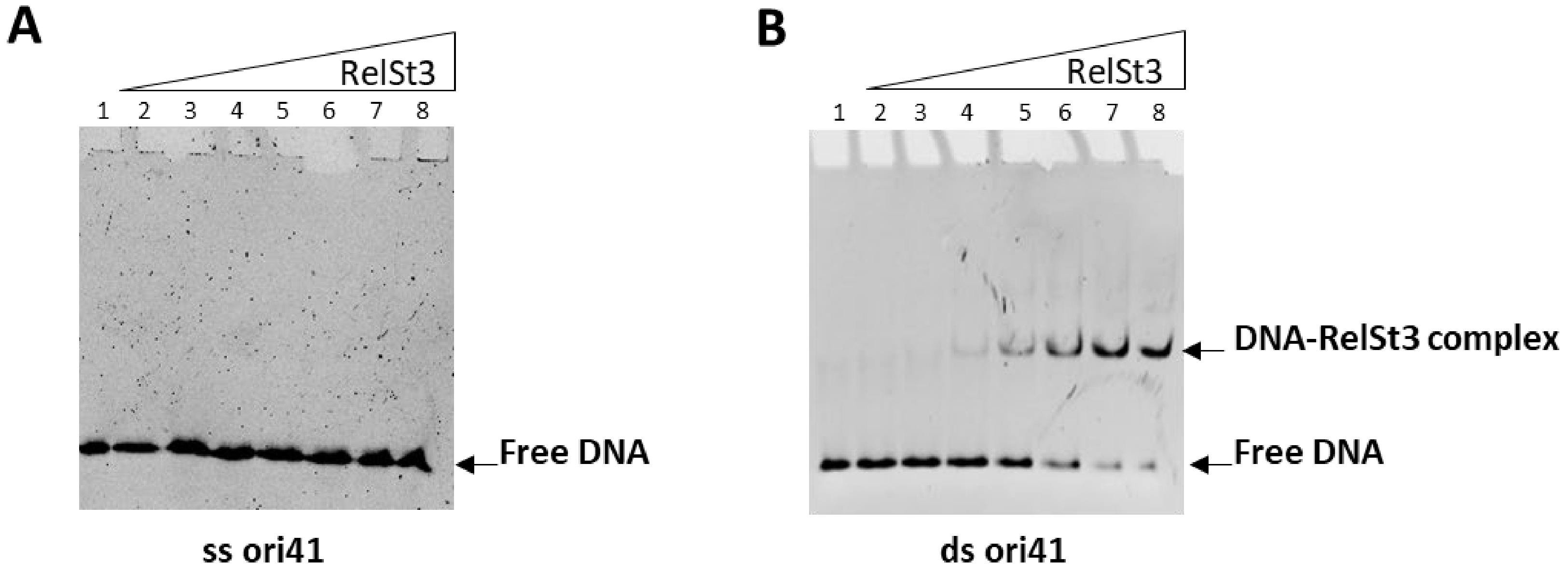

Supplementary Figure S3

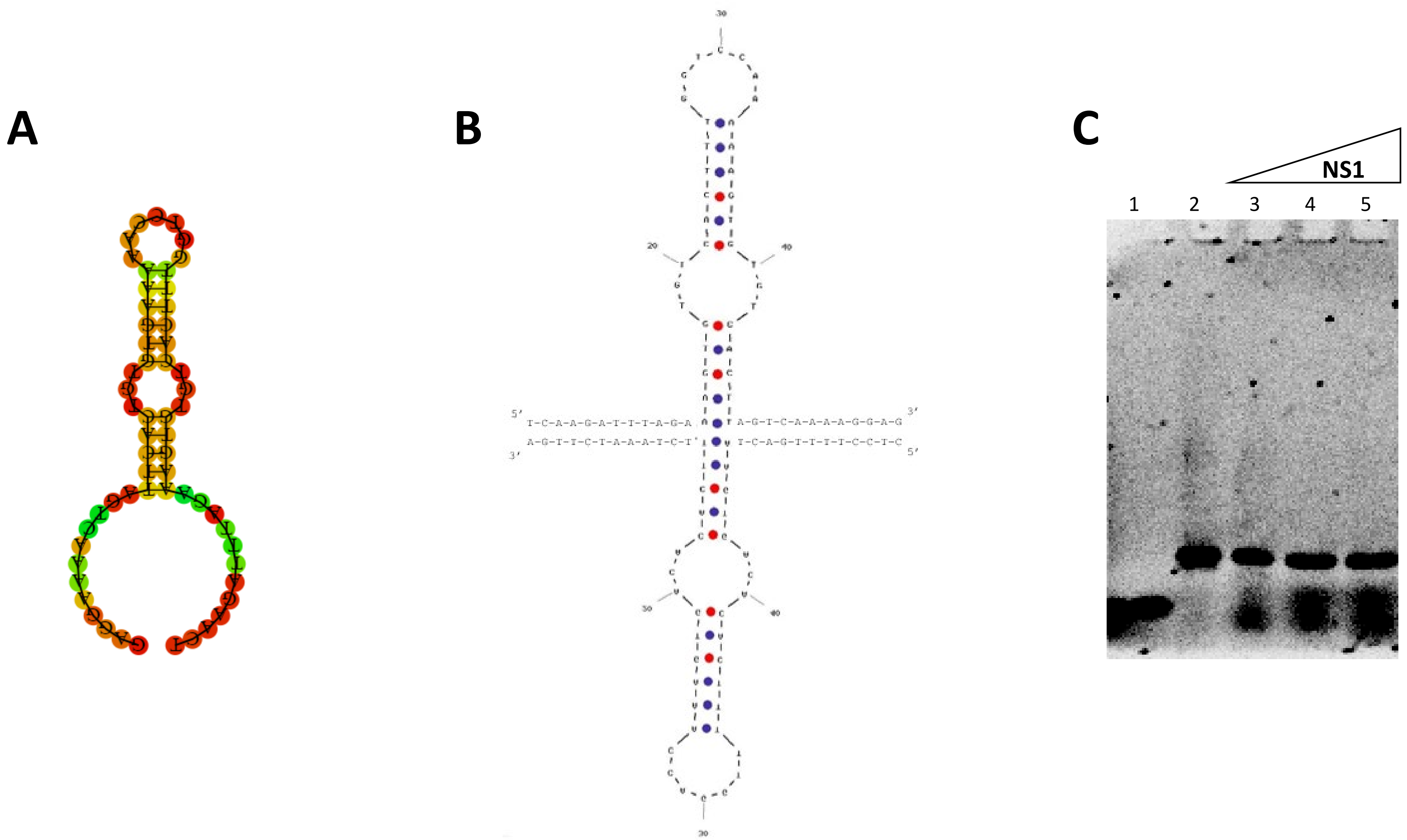

Supplementary Figure S4

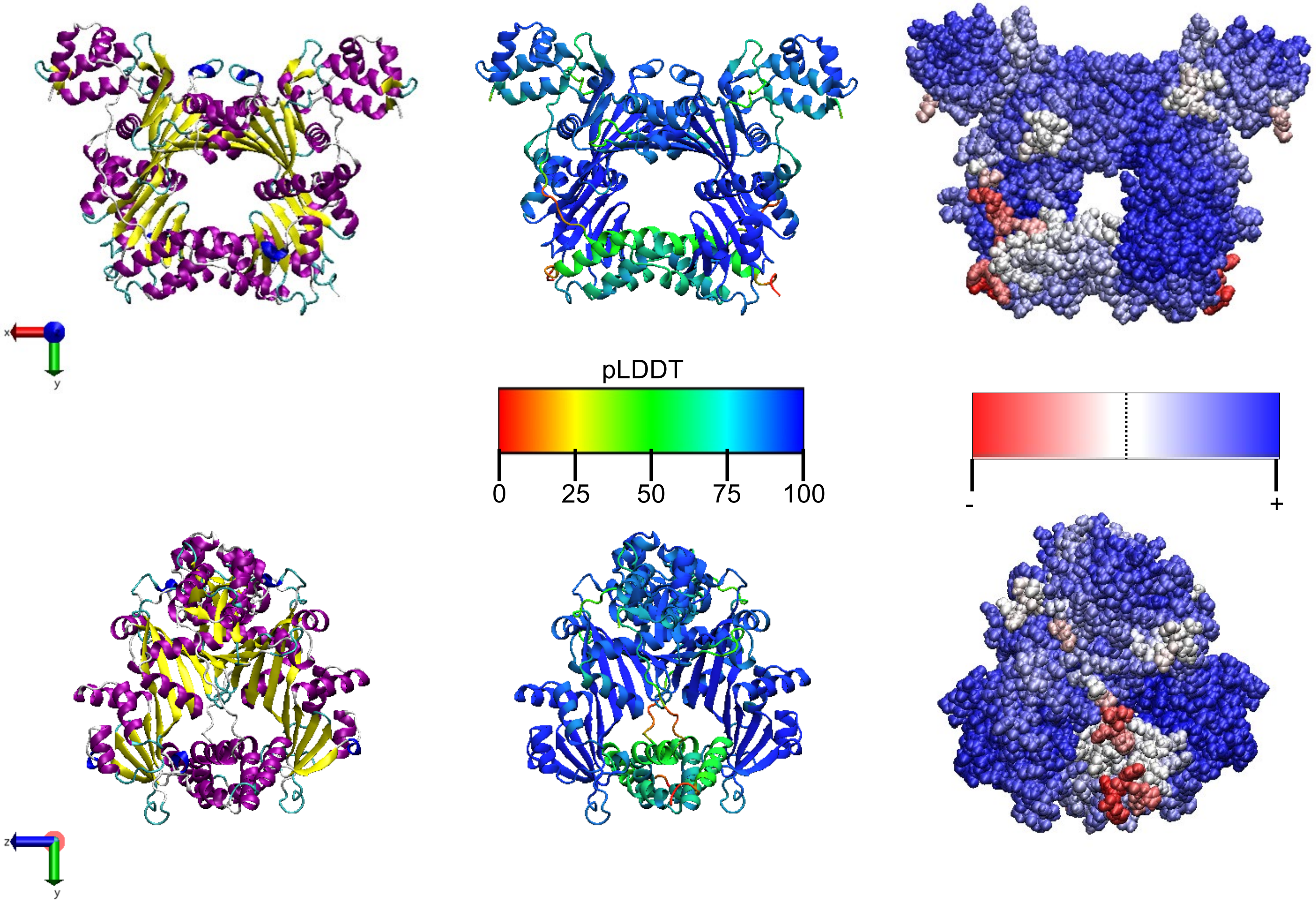

Supplementary Figure S5

A

nic site

bind site

ori50 5'* GAGACTTCAACCCCCGATTTCTAATAGGGGGGTTACATTTTCAAGATTTAGAAAGTGTGTCACTTTGGTCCAAAAAGTGTGTCACTTAGTCAAAAGGAG
AGTTCTAAATCTTTCACACAGTGAAACCAGGTTTTTCACACAGTGAATCAGTTTTCCTC 5'

22 nt ladder 5'* GAGACTTCAACCCCCGATTTCT

ori57 5'* AGGCGCAGACCGTAGCCCGAAGTTCCTAGGCCATGATGAGACTTCAACCCCCGATTTCT

ori56 5' AATAGGGGGGTTACATTTTCAAGATTTAGAAAGTGTGTCACTTTGGTCCAAAAAGTGTGTCACTTAGTCAAAAGGAG
AGTTCTAAATCTTTCACACAGTGAAACCAGGTTTTTCACACAGTGAATCAGTTTTCCTC 5'

B

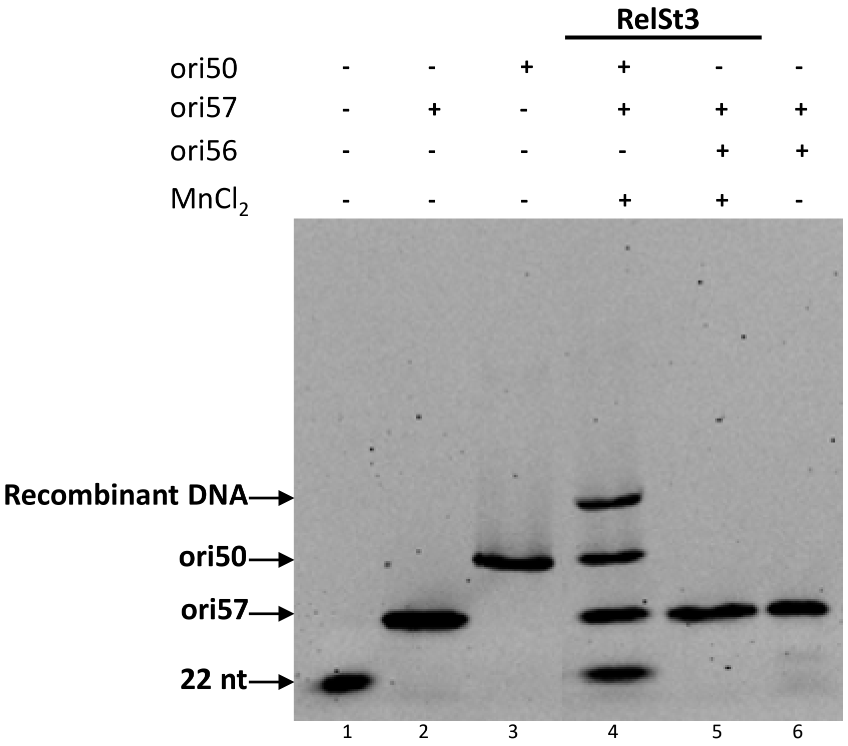

Supplementary Figure S7

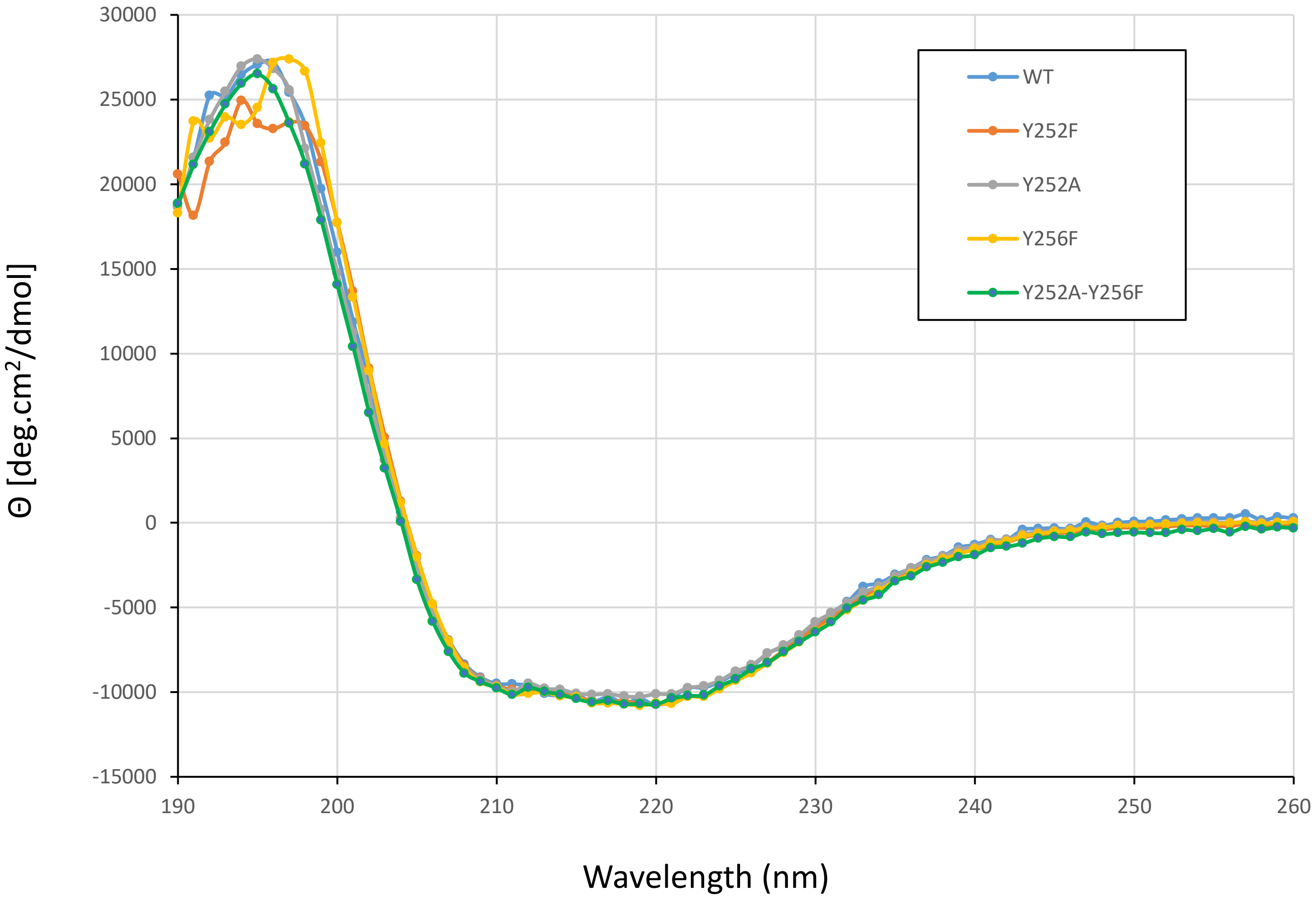

Supplementary Figure S8

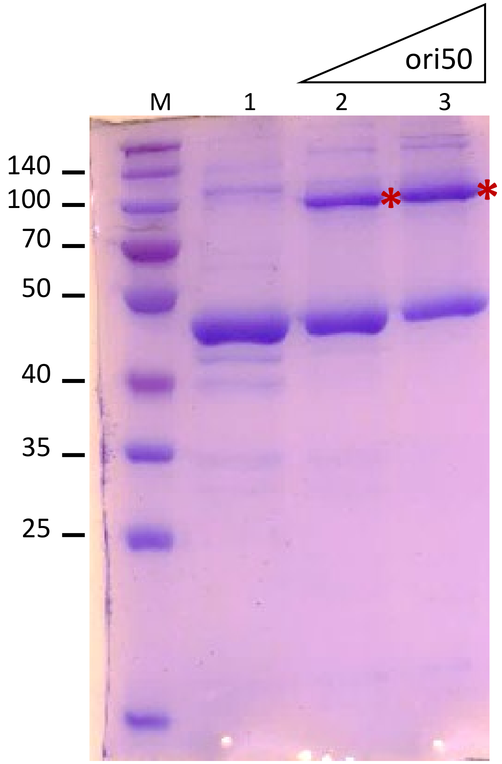

Supplementary Figure S10

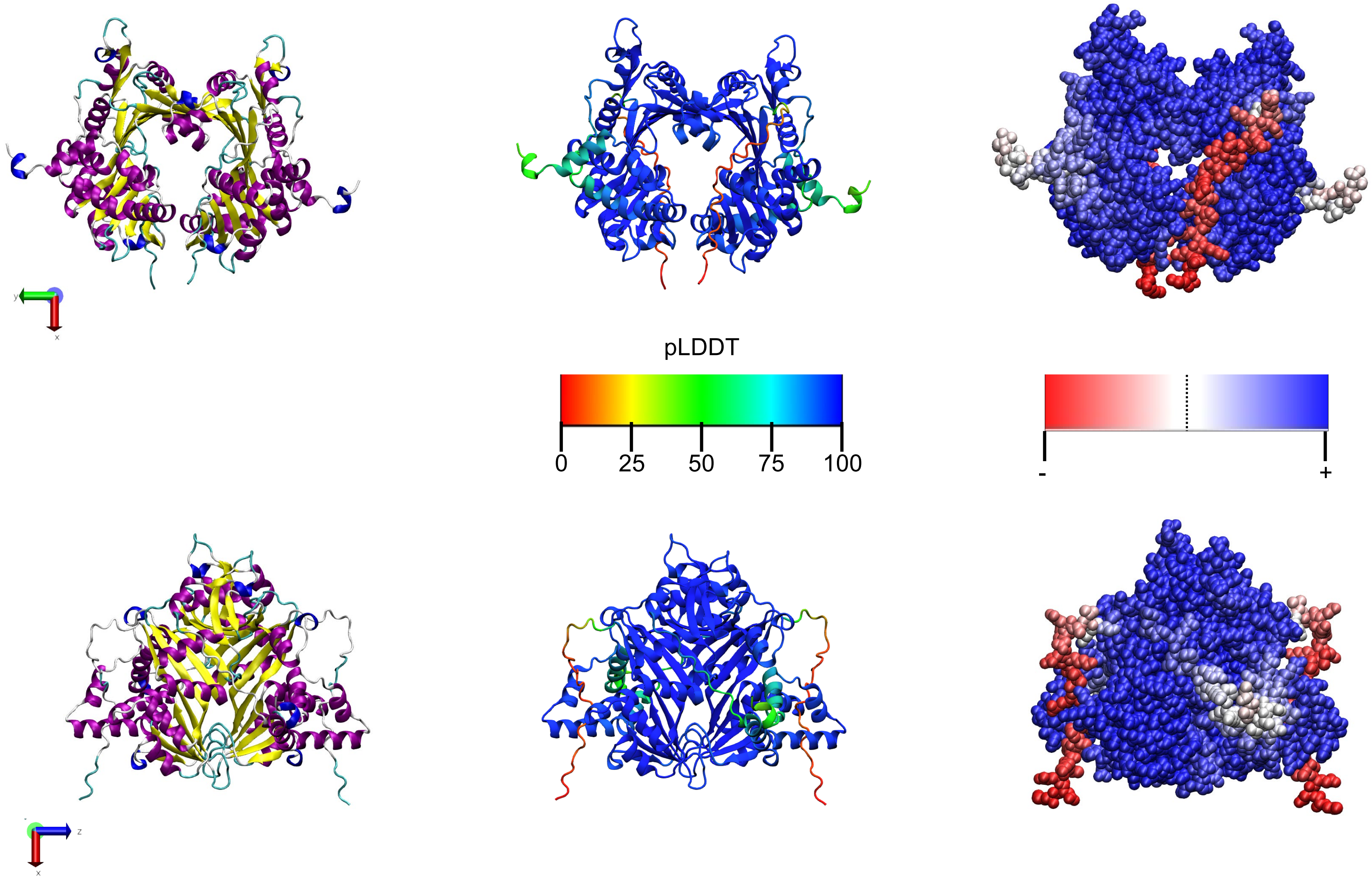

Supplement: gkac607_Supplemental_Files [file gkac607_supplemental_files.zip › Laroussi et al - Suppl Figures - Revised.pdf]
