# Supplementary material for: Exploration of DNA processing features unravels novel properties of ICE conjugation in Gram-positive bacteria
Source: Nucleic Acids Res. 2022 Jul 18;50(14):8127–42. doi: 10.1093/nar/gkac607 (PMC9371924; doi:10.1093/nar/gkac607)
Supplement: gkac607_Supplemental_Files [file gkac607_supplemental_files.zip › Laroussi et al - Suppl Mat - Revised.pdf]

# Supplementary Materials

## Exploration of DNA processing features unravels novel properties of ICE conjugation in Gram-positive bacteria

## Figures S6 and S9, Supplementary figure legends and Supplementary tables

```
                                         10        20        30        40        50        60        70
                                 ....|....|....|....|....|....|....|....|....|....|....|....|....|....|
Rel_ICESt3_Sthermophilus      1  -----------------MTKISPFQIKNFRKQTGLSQKAFAQAVNLPIRTYRSYESGERGLTIDKFRKLK 53
Rel_ICE515_Sagalactiae        1  -------------------------LKKFRKKTGLKQKEFALSSGLTLKSLRNYEQGKRKLTLEKYQEIK 45
Rel_Tn916_Efaecalis           1  ----------------MNEQTWLQHLKEKRLAYGLSQNRLAVATGITRQYLSDIETGKVKPSEDLQQSLW 54
Rel_ICEBs1_Bsubtilis          1  --------------------------------------------MDELKQPPHANRGVVIVKEK------ 20
Rel_Tn6009_Kpneumoniae        1  ---------------------------------------------------------------------- 1
Rel_Tn6202_Efaecalis          1  --------------------MTNQEIRQLRNRLGLSQQQFADKLHWSKSYLSMIETGKRTINKTAIERIN 50
Rel_ICE6013_Saureus           1  -------------------------------------------MTLKNQCTPLTNRGVERTNK------- 20
Rel_Tn6098_Llactis            1  -----------------------MELAKVRKSFGLSQNDIVKITGLSKSMVSMIDKGERQLNSESEQLLV 47
Rel_Nisin-sucrose transposon  1  ---------------------------------------MADLLGISLVYYRKMENGDRPLSKQFEEKIR 31
Rel_Tn5386                    1  ------------------------------------------MAGIRREHLSRIEAGRVTLTEDMKHKLL 28
Rel_ICECp1                    1  ------------------------------------------MAGISREHLNRIEAGKVTLTEDMQDKLM 28
Rel_EfaC2                     1  MILSHFSTKVVTFFGSRFKAVRGVDLKRYRKELKLKQQELASKLGIERSLISKIESGKRVISKELEQKII 70
Rel_ICE_SanC238_tRNAleu       1  --------------------MTNVELKRIRLELGFTQRKMATMIGYSYYNYRNIEQGQRKMTKEFEQTLF 50
Rel_ICE_Sdy12394_lysS         1  -----------------MTIIDGKQLRKFRASLGLKQKEFAEVAGLSLSSLKSYETGRREFTLEKFKEIK 53
Rel_ICE_SmiB6_guaA            1  ---------------MENEKIWIKELKEKRLAYGVSQNKLAVASHITRPYLSDIETGKAVPTQQVKEDLL 55
Rel_ICE_SparasFW213_ebfC      1  -----------------MDKISPFHIKNFRKQTGLSQKAFAQAVDLPTRTYRSYETGERGLTIDKFRELK 53
RepSTK1-4CIJ                  1  ---------------------------------------------------------------------- 1
RepC_pT181                    1  ----------------------------------------MYKNNHANHSNHLENHDLDNFSKTGYSNSR 30
RepD_pC221                    1  -------------------------------------------MSTENHSNYLQNKDLDNFSKTGYSNSR 27
RepN_pCW7                     1  ----------------------------------------MSKNNHANHSNHLENHDLDNFSKTGYSNSR 30
Rep_pRS2_Ooeni                1  ----------------------------------------MTKTSIEKPAPSNSRLSVKNVYT------- 23
Rep_pK214_Llactis             1  ---------------------------------------------------------------------- 1
Rep_pSP197_Staphpasteuri      1  ----------------------------------------------------MISAKVDQFTLTILPSLT 18
Rep_pBt1-3_Bthuringiensis     1  -----------------------------------------------------MEKHIEKLS-------- 9
Rep_pMC5_Exiguobacterium      1  -------------------------------------------------MKEGNDGGLVRHSVDKL---- 17
Rep_pMCCL4_Mcaseolyticus      1  ----------------------------------------MNKNELEKNWEPALSNSRLGV--------- 21
Rep_pQY003_Emundtii           1  ---------------------------------------MSKNGTIVNEWEKLTALSNRRLSVQ------ 25
Rep_pJS42_Efaecium            1  ----------------------------------------------MNEWEKLNRISNRRLKYQ------ 18
Rep_pF03-3_Lpentosus          1  ------------------------------------------------MFKLSFTLGGKGVFIM------ 16

                                         80        90       100       110       120       130       140
                                 ....|....|....|....|....|....|....|....|....|....|....|....|....|....|
Rel_ICESt3_Sthermophilus     54  EKLGYYQECHKNNLRAHIDYLRL--TEPSLRDLETFCE-------NFLFCHLSEFTDQ--ETRL---MNY 109
Rel_ICE515_Sagalactiae       46  SHFGYLVENDSSRLQVMIDYVRI--TLKDVRDLEFFCR-------NFLHCAFKEFQPF--ESKL---MNY 101
Rel_Tn916_Efaecalis          55  EALE--RFNPDAPLEMLFDYVRI--RFPT-TDVQQVVE-------NILQLKLSYFLHE--DYGF---YSY 107
Rel_ICEBs1_Bsubtilis         20  ------NEAVESPLVSMVDYIRV--SFKT-HDVDRIIE-------EVLHLSKDFMTEK--QSGF---YGY 69
Rel_Tn6009_Kpneumoniae        1  ---------------MLFDYVRI--RFPT-TDVQQVVE-------NILQLKLSYFLHE--DYGF---YSY 40
Rel_Tn6202_Efaecalis         51  QTFC--LEGGILPMQAKIDFLRI--RFKI-HAPDQVIE-------KVLRMNPEVFIYK--NYGF---NHY 103
Rel_ICE6013_Saureus          20  -----------SAVEAVVDWVQV--TEHI-DPISAVIE-------DVIGLPITLFKKR--NSGI---YFY 64
Rel_Tn6098_Llactis           48  DYL---HRKPKADITAMIDYLVI--RVKT-LNYKKFIN-------EVLKIPDYYFEQG--QSGG---NGY 99
Rel_Nisin-sucrose transposon 32  NSF-FKKRESSTVFVGTNDYTNI--RFQT-LNVREVVS-------KILGLNVENFQLN--EYNR---YQY 85
Rel_Tn5386                   29  EAVE--KFNPDNPMFLLFDYVRI--RFPT-MDIKHIIK-------DILKLNINYMLHE--DYGH---YKY 81
Rel_ICECp1                   29  EAVE--KFNPDAPMFLLFDYVRI--RFPT-LDIQHVIK-------DILKLNIDYMLHE--DYGH---YKY 81
Rel_EfaC2                    71  NVLN--LDGGHASVEAKIDFLRI--RFKT-LDVRTVIE-------KLLHMDMNWFTHE--SRGF---YHY 123
Rel_ICE_SanC238_tRNAleu      51  HFL---NRKSEAKLESTVDWLKI--RFKT-LDFKAIIT-------HVLKLRPTNFFHE--EKSL---YSY 102
Rel_ICE_Sdy12394_lysS        54  TNMGYSFSDSPHPLRLMIDYLRI--TFKNVRQLKEFVE-------SYLYVSFNEFTSQ--ETTM---MTY 109
Rel_ICE_SmiB6_guaA           56  NALE--RFNPDNPLEMLFDYVRI--RFPT-NDVAQIIG-------EVLRLNMDYMLHE--DFGY---YSY 108
Rel_ICE_SparasFW213_ebfC     54  ERLGYYQECDKNSLRAQIDYLRL--TEPRLKDLDTFCE-------NFLHCHLSEFTDQ--ETRL---MNY 109
RepSTK1-4CIJ                  1  ----------MSGLKPCVDWLQV--TEKTGQDSVKKCVEKLEKVFEILGLNEAEFLPL--KNGK---YGY 53
RepC_pT181                   31  LDA---HTVCISDPKLSFDAMTIVGNLNR-DNAQALS--------KFMSVEPQIRLWD--ILQ----TKF 82
RepD_pC221                   28  LSG---NFFTTPQPELSFDAMTIVGNLNK-TNAKKLS--------DFMSTEPQIRLWD--ILQ----TKF 79
RepN_pCW7                    31  LNR---HTMYTPEPKLSFDAMTIVGNLNK-NNAHKLS--------EFMSVEGQIRLWD--ILQ----TKF 82
Rep_pRS2_Ooeni               23  -----------SSIKWSIDRLTIVGELAP-FLPAGYTT-------NKISGEVKSRITI--EDMFKVFEHF 72
Rep_pK214_Llactis             1  --------MHPQNPKLSFDAMTIVGNLSR-DNAQKLS--------SFMSIEPQIRLWD--ILQ----TKF 47
Rep_pSP197_Staphpasteuri     19  NLEGFAH~~~~~~~~ISSDYSEYVRN--RFE--------------ELLQIVPTYSSAELMNGGV---RNY 61
Rep_pBt1-3_Bthuringiensis     9  -------------LSVCVDWLEF--TEVYGEEFESIC--------SFLGLDPTVFSKE--IDGFHKSYGY 54
Rep_pMC5_Exiguobacterium     17  ------SFVADPKDSGAIDQLEK--YLQE----------------KIMATRQLTKSHD--PYR----YMY 57
Rep_pMCCL4_Mcaseolyticus     21  ------QTTTTPLPKINFDRMTIIGDLPL-DRVEHMA--------EFLGNDPYVNLWE--KMN----NRF 70
Rep_pQY003_Emundtii          25  ------TLTTPLKLHWSIDRITIVGKLKE-NIYYHTQN-------DVLILDFEQLMRL--NEG----NGY 75
Rep_pJS42_Efaecium           18  ------SLTLPLKMQWSIDRITIVGKLKE-NIYYHTPN-------DVLILNFEQLMRL--NEG----NGY 68
Rep_pF03-3_Lpentosus         16  -----------RKVTVKIDRITVSGVLPN-WSLQDIHD----------------------ETGL---IPR 49

                                                   Motif N′
```

```
                                                     150       160       170       180       190       200       210
                                                ....|....|....|....|....|....|....|....|....|....|....|....|....|
Rel_ICESt3_Sthermophilus          110 THLWQR--GNIWIFD--FFDKSATNNYQTCLQLSGQGCR--EMELLLEHKGI-SWQT---FLQNILYAY- 168
Rel_ICE515_Sagalactiae            102 NHLWKR--GDIWIFD--FADKHETGNFQITVQLSGRGCR--QLELLMETEKF-TWHDWLSYLRNSYRDD- 163
Rel_Tn916_Efaecalis               108 SEHYAL--GDIFVLC---SHELDKG---VLVELKGRGCR--QFESYLLAQQR-SWYE---FFMDVLVAG- 162
Rel_ICEBs1_Bsubtilis              70  VGTYEL--DYIKVFY--SAPDDNRG---VLIEMSGQGCR--QFESFLECRKK-TWYD---FFQDCMQQG- 125
Rel_Tn6009_Kpneumoniae            41  SEHYAL--GDIFVLC---SHELDKG---VLVELKGRGCR--QFESYLLAQQR-SWYE---FFMDVLVAG- 95
Rel_Tn6202_Efaecalis              104 TETYCF--SEIFVFA--NPENLDMG---VMIELRGRGCR--EYELVLEEQQE-TWTE---FFWRLYETNL 160
Rel_ICE6013_Saureus               65  NRGYEF--SNIKLYY--SSDDESMG---IHLQLTGTGCR--EFEHHLQQLNK-TWQD---FFDKCLSVN- 120
Rel_Tn6098_Llactis                100 PFRVEY--GEIKVFY--HNENIDMG---ARIEFKGGACR--LFETFLEEQNR-TWQD---FLKDVINYSF 156
Rel_Nisin-sucrose transposon      86  PFYISY--GHINVYY--HDKDIKAG---VLIEMSGQACREMEYEFEYHQKQR-TWYD---FFNDCFLYAN 144
Rel_Tn5386                        82  TEHYYI--GDVVIYT---SADEEKG---VLLELKGKGCR--QFESYLLAQER-SWYD---FFMDALVEG- 136
Rel_ICECp1                        82  TEHYYL--GDVFVYT---SQDEEKG---TLLELKGKGCR--QFESYLLAQER-SWYD---FLMDALVEG- 136
Rel_EfaC2                         124 TETFSY--SSIRIFR--NPENVNMG---IMLDLSGEGCR--QLEEIFEEDNNRSWTE---FFRSLYDDDI 181
Rel_ICE_SanC238_tRNAleu           103 SDMVTY--GSIRVLYSHSEKKAEAG---TLIDLTGGGCR--EFELLLKQQGR-NWFS---FLHDVFLFAE 161
Rel_ICE_Sdy12394_lysS             110 NHLYKR--GDIWIFD--YFDKEERSNYQITLQLSGQGCR--QMELILEREGI-SWRD---FLEKMLYER- 168
Rel_ICE_SmiB6_guaA                109 PEHYRF--GDIVVLV---SHDVSKG---VLLELKGKGCR--QFENFLLAQHR-SWYD---FFQDCMEHK- 163
Rel_ICE_SparasFW213_ebfC          110 THLWQR--GNIWIFD--FFDKSVTNDYQTCLQLSGQGCR--ELELLLEDKGI-TWQI---FLQNILYSY- 168
RepSTK1-4CIJ                      54  KQGVAFQGNPVLAVY--YDGADDMG---IHVEMTGQGCR--LFELHTSI----NWYE--LFYRLVYEYE- 109
RepC_pT181                        83  KAKALQ--EKVYIEY----DKVKAD------SWDRRNMR-IEFNPNKLTRDEMIWLK--QNIISYMEDD- 136
RepD_pC221                        80  KAKALQ--EKVYIEY----DKVKAD------SWDRRNMR-VEFNPNKLTHEEMLWLK--QNIIDYMEDD- 133
RepN_pCW7                         83  KAKALQ--EKVYIEY----DKVKAD------TWDRRNMR-VEFNPNKLTHEEMLWLK--QNIIDYMEDD- 136
Rep_pRS2_Ooeni                    73  GHCRLS--GNGYQMI---TDDGENVAYFERLKFDKNKGR-LDFNPNKLDSFFENGLK--QFISDLFITP- 133
Rep_pK214_Llactis                 48  KAKALQ--EKVYIEY----DKVKAD------TWDRRNMR-VEFNPNKLTHEEMIWLK--QNIIDYMEDD- 101
Rep_pSP197_Staphpasteuri          62  DTVYSYGFEKAKIFFKYSSSNVSNG---ISIEFKARALR--EYLKAYRIKKS-DSMD--VHRILSLLAD- 122
Rep_pBt1-3_Bthuringiensis         55  LSRYSF--EEIHILM----HGADNR---SRIIMSGSGCR--WFETLSSV----GWYG---LFDRIAFAD- 105
Rep_pMC5_Exiguobacterium          58  ILPMKL--GTVSIAD----KNAKLG----------KRLR-LEFNPNRATSDDVRQMY--INIIATMKYP- 107
Rep_pMCCL4_Mcaseolyticus          71  KGKALN--EKVYIEH----DRLKAD------AWNRRNFR-IEFNPNNLSDDEKLWIR--ENLSSVLENV- 124
Rep_pQY003_Emundtii               76  LKAVG---NNGWQLLDQYEENIAYI---EILKWQEGKGR-IDFNPSKINQFLAGSMK--NFIHDLFLEP- 135
Rep_pJS42_Efaecium                69  LKAVG---NNGWQLLDQHEENIAYV---EILKFHEGKGR-IDFNPNKINQFLSGSMK--NFIHDLFLEP- 128
Rep_pF03-3_Lpentosus              50  D-------GAMFLEREDKDGNTENMAFMAESPFQREHWR-LDFNPANLTADETAVLG---RVINNMEQA- 107
```

Motif N″

```
                                                     220       230       240       250       260       270       280
                                                ....|....|....|....|....|....|....|....|....|....|....|....|....|
Rel_ICESt3_Sthermophilus          168 ---------------QDVRVKRLDIALDELYKGYGHEEEHIQIPKLIDKLYAK---EIVLDTIRKWNITG 220
Rel_ICE515_Sagalactiae            163 -----------------MNVTRFDIAIDELYLGKDRENEQFHLSDMISKYYRH---ELDFESLRTWNYIG 213
Rel_Tn916_Efaecalis               162 -----------------GVMKRLDLAIND-KTGI------LNIPVLTEK-CQQ---EECISVFRSFKSYR 204
Rel_ICEBs1_Bsubtilis              125 -----------------GSFTRFDLAIDD-KKTY------FSIPELLKK-AQK---GECISRFRKSDFNG 167
Rel_Tn6009_Kpneumoniae            95  -----------------GVMKRLDLAIND-KTGI------LNIPVLTEK-CQQ---EECISVFRSFKSYR 137
Rel_Tn6202_Efaecalis              161 FDNHR---------MIDTKITRIDLALDEQVSLLYPS---YDLFELKAK-YEQ---GLVDTTFRNFDFTG 214
Rel_ICE6013_Saureus               120 -----------------ANFTRIDIAIDD-YKTY------LKVPLLIKK-AEK---AECVSRFRAGSAIN 162
Rel_Tn6098_Llactis                157 EATRINGVDDPIEARKFLKFKRLDIALDERFNEKGN----YNLMALWEK-VRK---GQIEMKLKGFRPEE 218
Rel_Nisin-sucrose transposon      145 KKAPEN--------DDFVKITRFDLALDEQYNPQEGN---FDLFKLLTS-ARE---GRWNGRKQNYSAVL 199
Rel_Tn5386                        136 -----------------GVMKRLDLAIND-RTGL------LDIPELIKK-CEN---EECISKFRSFKNYG 178
Rel_ICECp1                        136 -----------------GVMKRLDLAIND-RAGI------LDIPDLTAK-CNR---EECVSLFRSFKSYA 178
Rel_EfaC2                         182 FGQGI---------LVDTKITRIDIALDELIVKGQEN---FDLYVLKEK-MEQ---GLVDTTFKNFDFSG 235
Rel_ICE_SanC238_tRNAleu           162 QERKDRPL------EDFLAFPRFDIALDELYKKSGN----LDLFDIKNRIFDN---KIIMKKLKTFTAIE 218
Rel_ICE_Sdy12394_lysS             168 ---------------SDMKVTRIDLALDELYRGYDQEETHFHLSDMINKVYQH---LVTFDRLRTWSHIG 220
Rel_ICE_SmiB6_guaA                163 -----------------GIFKRLDLAIND-KTGI------LNIPDLAKK-CKQ---EECISVFRSFKNYR 205
Rel_ICE_SparasFW213_ebfC          168 ---------------EDVRVKRLDIALDELYKGYGHEDEQIQIPKLIDKLYSK---EIVLDTIKKWNITG 220
RepSTK1-4CIJ                      109 -----------------VNITRLDVAVDD-FKGY------FKINTLVKK-LKD---DEVTSRFKKARHIE 151
RepC_pT181                        136 ------------------GFTRLDLA-------------FDFEDDLSDYY-----AMSDKAVKKTIFYG 169
RepD_pC221                        133 ------------------GFTRLDLA-------------FDFEDDLSDYY-----AMTDKAVKKTIFYG 166
RepN_pCW7                         136 ------------------GFTRLDLA-------------FDFEYDLSDYY-----AMTDKSVKKTIFYG 169
Rep_pRS2_Ooeni                    133 ------------------HFSRADIAADI----------FDVPDEKVVSYRL---GEPVG---NTFFYG 168
Rep_pK214_Llactis                 101 ------------------GFTRLDLA-------------FDFEDDLSDYY-----AMTDKAVKKTIFYG 134
Rep_pSP197_Staphpasteuri          122 --------------EFNIRLSRLDIAIDF----------VDEDVLVTKIYDDLLSEELIIKNKGNRIIS 167
Rep_pBt1-3_Bthuringiensis         105 --EHG---------FSWIKVDRLDIAIDD-FVGY------FSVKKLKSK-IKR---RECLSRWKTSFVVE 153
Rep_pMC5_Exiguobacterium          107 ------------------QLTRVDLA-------------FDYAENLSDI------RWVDKKGRPSSLYR 139
Rep_pMCCL4_Mcaseolyticus          124 ------------------GFTRLDLA-------------FDFEEDLSDFF-----VMSDNALKKTVFYG 157
Rep_pQY003_Emundtii               135 ------------------HFSRADIACDI----------IDVPDEFITQYRV---VDPVS----FKPIY 169
Rep_pJS42_Efaecium                128 ------------------HFSRADIACDI----------VDIPDDFVSQYRV---VDPVS----FKPIY 162
Rep_pF03-3_Lpentosus              107 ------------------HFTRLDIA-------------FDVFN-----------DDLAMKYRVYRFNT 134
```

Motif I

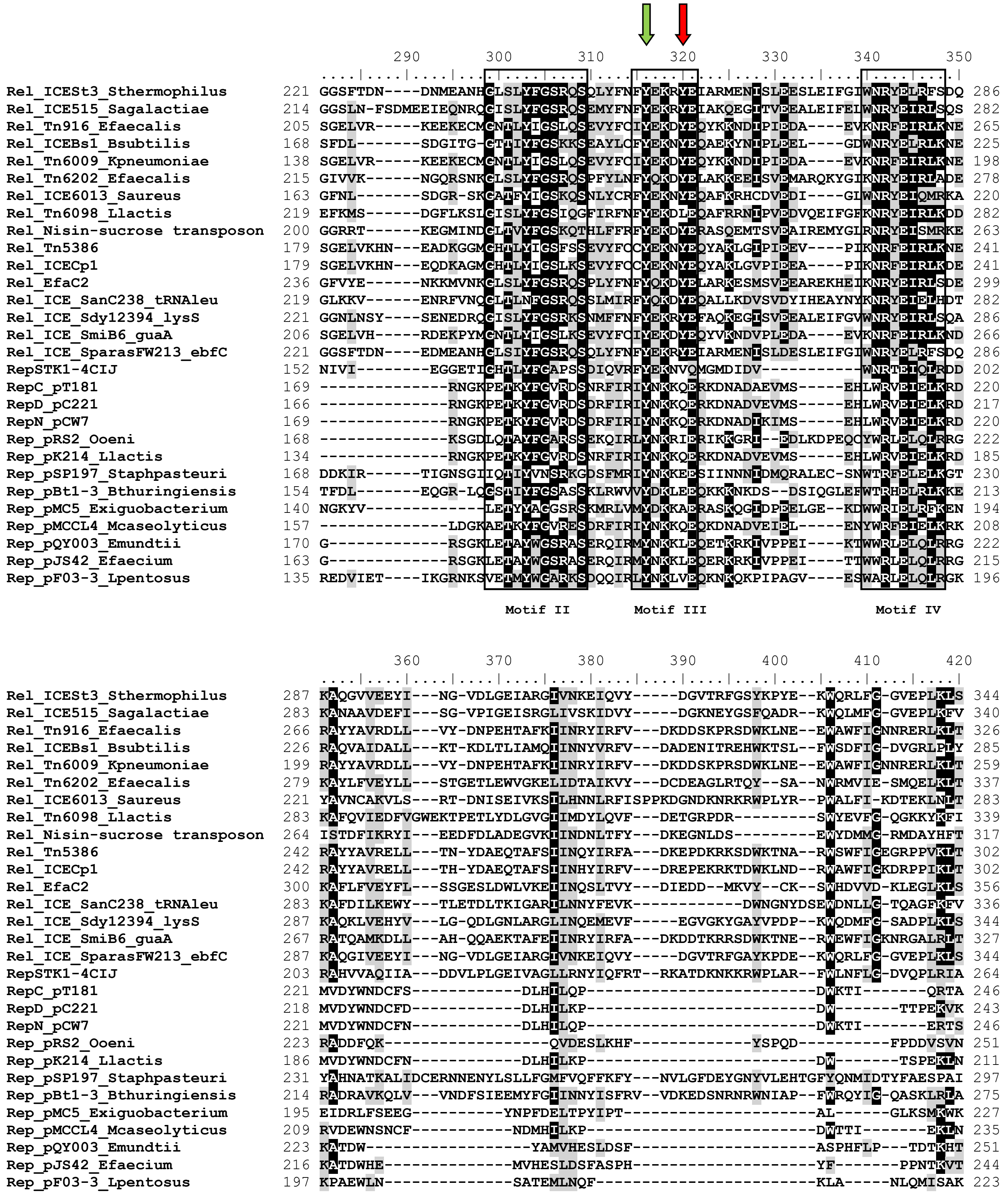

290 300 310 320 330 340 350
Rel_ICESt3_Sthermophilus 221 GGSFTDN----DNMEANHGLSLYFGSRQSQLYFNFYEKRYEIARMENISLEESLEIFGIWNRYELRFSDQ 286
Rel_ICE515_Sagalactiae 214 GGSLN-FSDMEEIEQNRQGISLYFGSRQSEMYFNFYEKRYEIAKQEGITVEEALEIFEIWNRYEIRLSQS 282
Rel_Tn916_Efaecalis 205 SGELVR-----KEEKECMGNTLYIGSLQSEVYFCIYEKDYEQYKKNDIPIEDA----EVKNRFEIRLKNE 265
Rel_ICEBs1_Bsubtilis 168 SFDL-------SDGITG-GTTIYFGSKKSEAYLCFYEKNYEQAEKYNIPLEEL----GDWNRYELRLKNE 225
Rel_Tn6009_Kpneumoniae 138 SGELVR-----KEEKECMGNTLYIGSLQSEVYFCIYEKDYEQYKKNDIPIEDA----EVKNRFEIRLKNE 198
Rel_Tn6202_Efaecalis 215 GIVVK------NGQRSNKGLSLYFGSRQSPFYLNFYQKDYELAKKEEISVEMARQKYGIKNRYEIRLADE 278
Rel_ICE6013_Saureus 163 GFNL-------SDGR-SKGATFYIGSKQSNLYCRFYEKNYEQAFKRHCDVEDI----GIWNRYEIQMRKA 220
Rel_Tn6098_Llactis 219 EFKMS------DGFLKSIGISLYFGSIQGFIRFNFYEKDIEQAFRRNIPVEDVQEIFGFKNRYEIRLKDD 282
Rel_Nisin-sucrose transposon 200 GGRRT------KEGMINDGLTVYFGSKQTHLFFRFYEKDYERASQEMTSVEAIREMYGIRNRYEISMRKE 263
Rel_Tn5386 179 SGELVKHN---EADKGGMGHTLYIGSFSSEVYFCCYEKNYEQYAKLGIPIEEV----PIKNRFEIRLKNE 241
Rel_ICECp1 179 SGELVKHN---EQDKAGMGHTLYIGSLKSEVYFCCYEKNYEQYAKLGVPIEEA----PIKNRFEIRLKDE 241
Rel_EfaC2 236 GFVYE------NKKMVNKGLSLYFGSRQSPLYFNFYQKDYELARKESMSVEEAREKHEIKNRYEIRLSDE 299
Rel_ICE_SanC238_tRNAleu 219 GLKKV------ENRFVNQGLTLNFGSRQSSLMIRFYQKDYEQALLKDVSVDYIHEAYNYKNRYEIELHDT 282
Rel_ICE_Sdy12394_lysS 221 GGNLNSY----SENEDRQGISLYFGSRKSNMFFNFYEKRYEFAQKEGISVEEALEIFGVWNRYEIRLSQA 286
Rel_ICE_SmiB6_guaA 206 SGELVH-----RDEKPYMGNTLYIGSLKSEVYFCIYEKDYEQYVKNDVPLEDA----EVKNRFEIRLKND 266
Rel_ICE_SparasFW213_ebfC 221 GGSFTDN----EDMEANHGLSIYFGSRQSQLYFNFYEKRYEIARMENISLDESLEIFGIWNRYELRFSDQ 286
RepSTK1-4CIJ 152 NIVI--------EGGETIGHTLYFGAPSSDIQVRFYEKNVQMGMDIDV------------WNRTEIQLRDD 202
RepC_pT181 169 --------------RNGKPETKYFGVRDSNRFIRIYNKKQERKDNADAEVMS-----EHLWRVEIELKRD 220
RepD_pC221 166 --------------RNGKPETKYFGVRDSDRFIRIYNKKQERKDNADVEVMS-----EHLWRVEIELKRD 217
RepN_pCW7 169 --------------RNGKPETKYFGVRDSDRFIRIYNKKQERKDNADIKIMS-----EHLWRVEIELKRD 220
Rep_pRS2_Ooeni 168 --------------KSGDLQTAYFGARSSEKQIRLYNKRIERIKKGRI--EDLKDPEQQYWRLELQLRRG 222
Rep_pK214_Llactis 134 --------------RNGKPETKYFGVRDSNRFIRIYNKKQERKDNADVEVMS-----EHLWRVEIELKRD 185
Rep_pSP197_Staphpasteuri 168 DDKIR------TIGNSGIIQTIYVNSRKGDSFMRIYNKKEESIINNNIDMQRALEC-SNWTRFELELKGT 230
Rep_pBt1-3_Bthuringiensis 154 TFDL-------EQGR-LQGSTIYFGSASSKLRWVVYDKLEEQKKKNKDS--DSIQGLEEWTRHELRLKKE 213
Rep_pMC5_Exiguobacterium 140 NGKYV------------LETYYAGGSRSKMRLVMYDKKAERASKQGIDPEELGE--KDWWRIELRFKEN 194
Rep_pMCCL4_Mcaseolyticus 157 --------------LDGKAETKYFGVRESDRFIRIYNKKQEQKDNADVEIEL-----ENYWRFEIELKRK 208
Rep_pQY003_Emundtii 170 G-------------RSGKLETAYWGSRASERQIRMYNKKLEQETKRKIVPPEI----KTWWRLELQLRRG 222
Rep_pJS42_Efaecium 163 G-------------RSGKLETAYWGSRASERQIRMYNKKLEQERKRKIVPPEI----TTWWRLELQLRRG 215
Rep_pF03-3_Lpentosus 135 REDVIET----IKGRNKSVETMYWGARKSDQQIRLYNKLVEQKNKQKPIPAGV----ESWARLELQLRGK 196
Motif II
Motif III
Motif IV
360 370 380 390 400 410 420
Rel_ICESt3_Sthermophilus 287 KAQGVVEEYI---NG-VDLGEIARGIVNKEIQVY-----DGVTRFGSYKPYE--KWQRLFG-GVEPLKLS 344
Rel_ICE515_Sagalactiae 283 KANAAVDEFI---SG-VPIGEISRGLIVSKIDVY-----DGKNEYGSFQADR--KWQLMFG-GVEPLKFV 340
Rel_Tn916_Efaecalis 266 RAYYAVRDLL---VY-DNPEHTAFKIINRYIRFV---DKDDSKPRSDWKLNE--EWAWFIGNNRERLKLT 326
Rel_ICEBs1_Bsubtilis 226 RAQVAIDALL---KT-KDLTLIAMQIINNYVRFV---DADENITREHWKTSL--FWSDFIG-DVGRLPLY 285
Rel_Tn6009_Kpneumoniae 199 RAYYAVRDLL---VY-DNPEHTAFKIINRYIRFV---DKDDSKPRSDWKLNE--EWAWFIGNNRERLKLT 259
Rel_Tn6202_Efaecalis 279 KAYLFVEYLL---STGETLEWVGKELIDTAIKVY---DCDEAGLRTQY--SA--NWRMVIE-SMQELKLT 337
Rel_ICE6013_Saureus 221 YAVNCAKVLS---RT-DNISEIVKSILHNNLRFISPPKDGNDKNRKRWPLYR--PWALFI-KDTEKLNLT 283
Rel_Tn6098_Llactis 283 KAFQVIEDFVGWEKTPETLYDLGVGIIMDYLQVF---DETGRPDR---------SWYEVFG-QGKKYKFI 339
Rel_Nisin-sucrose transposon 264 ISTDFIKRYI---EEDFDLADEGVKIINDNLTFY---DKEGNLDS---------EWYDMMG-RMDAYHFT 317
Rel_Tn5386 242 RAYYAVRELL---TN-YDAEQTAFSIINQYIRFA---DKEPDKRKSDWKTNA--RWSWFIGEGRPPVKLT 302
Rel_ICECp1 242 RAYYAVRELL---TH-YDAEQTAFSIINHYIRFV---DREPEKRKTDWKLND--RWAWFIGKDRPPIKLT 302
Rel_EfaC2 300 KAFLFVEYFL---SSGESLDWLVKEIINQSLTVY---DIEDD--MKVY--CK--SWHDVVD-KLEGLKLS 356
Rel_ICE_SanC238_tRNAleu 283 KAFDILKEWY---TLETDLTKIGARILNNYFEVK-----------DWNGNYDSEWDNLLG-TQAGFKFV 336
Rel_ICE_Sdy12394_lysS 287 KAQKLVEHYV---LG-QDLGNLARGLINQEMEVF-----EGVGKYGAYVPDP--KWQDMFG-SADPLKLS 344
Rel_ICE_SmiB6_guaA 267 RATQAMKDLL---AH-QQAEKTAFEIINRYIRFA---DKDDTKRRSDWKTNE--RWEWFIGKNRGALRLT 327
Rel_ICE_SparasFW213_ebfC 287 KAQGIVEEYI---NG-VDLGEIARGIVNKEIQVY-----DGVTRFGAYKPDE--KWQRLFG-GVEPLKLS 344
RepSTK1-4CIJ 203 RAHVVAQIIA---DDVLPLGEIVAGLLRNYIQFRT--RKATDKNKKRWPLAR--FWLNFLG-DVQPLRIA 264
RepC_pT181 221 MVDYWNDCFS------------DLHILQP-----------------------DWKTI-------QRTA 246
RepD_pC221 218 MVDYWNDCFD------------DLHILKP-----------------------DW-------TTPEKVK 243
RepN_pCW7 221 MVDYWNDCFN------------DLHILQP-----------------------DWKTI-------ERTS 246
Rep_pRS2_Ooeni 223 RADDFQK------------------QVDESLKHF-------------YSPQD----------FPDDVSVN 251
Rep_pK214_Llactis 186 MVDYWNDCFN------------DLHILKP-----------------------DW-------TSPEKLN 211
Rep_pSP197_Staphpasteuri 231 YAHNATKALIDCERNNENYLSLLFGMFVQFFKFY---NVLGFDEYGNYVLEHTGFYQNMIDTYFAESPAI 297
Rep_pBt1-3_Bthuringiensis 214 RADRAVKQLV---VNDFSIEEMYFGIINNYISFRV--VDKEDSNRNRWNIAP--FWRQYIG-QASKLRLA 275
Rep_pMC5_Exiguobacterium 195 EIDRLFSEEG----------YNPFDELTPYIPT-------------------AL-----GLKSMKWK 227
Rep_pMCCL4_Mcaseolyticus 209 RVDEWNSNCF------------NDMHILKP-----------------------DWTTI-------EKLN 235
Rep_pQY003_Emundtii 223 KATDW-----------------YAMVHESLDSF-------------------ASPHFLP---TDTKHT 251
Rep_pJS42_Efaecium 216 KATDWHE---------------MVHESLDSFASPH--------------------YF-------PPNTKVT 244
Rep_pF03-3_Lpentosus 197 KPAEWLN---------------SATEMLNQF-----------------------KLA-----NLQMISAK 223

```
                                        430       440       450       460       470       480       490
                                   ....|....|....|....|....|....|....|....|....|....|....|....|....|....|
Rel_ICESt3_Sthermophilus      345 TSPQPYSIERTIR---WLTYQVANSLALV-SEADKIMQ-------TEYMKMIQNS--GEITDRGEAILRL 401
Rel_ICE515_Sagalactiae        341 TKPEAYSIERTLR---WLSDSVSPSLAMI-REYDMIVD-------GDYLQTILNS--GEVNERGEKILDS 397
Rel_Tn916_Efaecalis           327 TKPEPYSFQRTLN---WLSHQVAPTLKVA-IKLDEINQ-------TQVVKDILDH--AKLTDRHKQILKQ 383
Rel_ICEBs1_Bsubtilis          286 VKPQKDFYQKSRN---WLRNSCAPTMKMV-LEADEHLG-------KTDLSDMIAE--AELADKHKKMLDV 342
Rel_Tn6009_Kpneumoniae        260 TKPEPYSFQRTLN---WLSHQVAPTLKVA-IKLDEINQ-------TQVVKDILDH--AKLTDRHKQILKQ 316
Rel_Tn6202_Efaecalis          338 MKGEKPSYEKSLR---WLSNYLAPTLKKI-WIMDQTFG-------TDELMTRIKQ--AELKEKDQEELAK 394
Rel_ICE6013_Saureus           284 TRPTLKSIEDNLD---WLCKQVATTLDTV-LTAESMAQSEGLLTDTDFLDKILAH--SQFNDEHTNRINH 347
Rel_Tn6098_Llactis            340 TQPKIESLEKRRA---WFDRQLKRNLYIE-ARISRETG-------RSYVEELISE--YDPDDEDEKIIRR 396
Rel_Nisin-sucrose transposon  318 VRPEAPDLNRKYT---WFERGGPVSTYLLLKKAEELTG-------ESRLEEIFNE--AELTERQEKFLKE 375
Rel_Tn5386                    303 TKPEPYTLERTLN---WLQRQVAPTLKML-KKIDKDNQ-------TDYLETIEQQ--ARLTERHHQIIKQ 359
Rel_ICECp1                    303 TDPEPYTLERTLG---WISRQVAPTLKML-KKIDAGNS-------TSYLKEIEDN--AKLTEKHLQIIRQ 359
Rel_EfaC2                     357 VQGEKPSIEKTLR---WLSNYLAPSLKMI-KEIDNLLG-------TNELMERIDL--AELKEKHEEIIEM 413
Rel_ICE_SanC238_tRNAleu       337 TRPRQINYARTKH---WVTKQVSSALKLL-KIVDMVYQ-------TDELSEIISE--AYLSNNHTKLAEE 393
Rel_ICE_Sdy12394_lysS         345 VQPEPYSIDRTVR---WLLYQVSNSLSFV-EEADKIMN-------TQYLEMIQNT--GKLTDRMETELKY 401
Rel_ICE_SmiB6_guaA            328 TQPEPYSFERTLN---WLHHQVAPTLKIA-SILDVLNG-------TTIISTMIQE--AKLTEKHEKLIEQ 384
Rel_ICE_SparasFW213_ebfC      345 TNPQPYSIERTIR---WLTYQVANSLALV-TEADKILQ-------TEYMKMIQNS--GEITDRGKAMLRL 401
RepSTK1-4CIJ                  265 KQMPKTSIEKKYR---WIDSQVSKSFFMIYYCLNEEEK-------QRFIDDVLAEGASKLTKADLQVINQ 324
RepC_pT181                    247 DRAIVFMLLSDEEE--WGKLHRNSRTKYK-NLIKEIS--------PVDLTDLMKS---TLKANEKQLQKQ 302
RepD_pC221                    244 EQAMVYLLLNEEGT--WGKLERHAKYKYK-QLIKEIS--------PIDLTELMKS---TLKENEKQLQKQ 299
RepN_pCW7                     247 DRAMVFMLLNDEEE--WGKLERRTKNKYK-KLIKEIS--------LIDLTDLMKS---TLKANEKQLQKQ 302
Rep_pRS2_Ooeni                252 SRIFLKGLLADPDN--WARISRPTKSKYR-QIEERVAS-------SDKLTIELKK---TFSQQNKKIEKE 308
Rep_pK214_Llactis             212 EQAMVYMLIHEEGK--WGELNKRTKYKYK-KIIKEIS--------PIDLTEIMKL---TLRENEKQLQKQ 267
Rep_pSP197_Staphpasteuri      298 DGHKTNKADEFGRK--YGNLFNNGTMTFF-RMYKEVYG-------ENELDELLNKIKEDIENIELNKDHE 357
Rep_pBt1-3_Bthuringiensis     276 EALPVANVERTFK---WLSHSVSKKLYLLSEIFSQDDD-----TKKEFASYLENKGKQKITKGDLDLIEN 337
Rep_pMC5_Exiguobacterium      228 DRCVVMALMNEEGESIFGEMDAKSRAKYK-KMLLEYTM-------PT--PVDVRE---DFEQQKNDLLAQ 284
Rep_pMCCL4_Mcaseolyticus      236 ERAIVCMLLTHENE--WGKLARHTKYKYR-KMIKEIS--------PIDLTDLMRE---CLKKEESRLQKE 291
Rep_pQY003_Emundtii           252 DRIMIAGLISDQNL--WAGLARHTKYKYR-NLLKQESQ-------NDELTNHLRE---SFAESADELKTE 308
Rep_pJS42_Efaecium            245 ERIMVKGLLYDHSE--WGNLAKNTKKKYR-DLLKQESQ-------NDELTNHLRE---SFAESADDLKRE 301
Rep_pF03-3_Lpentosus          224 DRAILYALTHDIIE--WQEISVATRSKYR-KMIKQSDGF------ETELADEMKQ---VLADNLDELQAE 281

                                        500       510       520       530
                                   ....|....|....|....|....|....|....|....|..
Rel_ICESt3_Sthermophilus      402 LKTNKHYEH--------------------------------- 410
Rel_ICE515_Sagalactiae        398 IKAS-------------------------------------- 401
Rel_Tn916_Efaecalis           384 Q---SVKEQDVITTKK-------------------------- 396
Rel_ICEBs1_Bsubtilis          343 Y---MADVADMVV----------------------------- 352
Rel_Tn6009_Kpneumoniae        317 Q---SVKEQDVITTKK-------------------------- 329
Rel_Tn6202_Efaecalis          395 L---TTTIKELLIQEEEEVVSSKTVSVTQQEVEQLLAQFLFE 433
Rel_ICE6013_Saureus           348 Y-LEALKQKKHLSKDKC------------------------- 363
Rel_Tn6098_Llactis            396 -DVERIKNRDFEHEARSTPEGQQAFLKALGK----------- 425
Rel_Nisin-sucrose transposon  376 FRMIRGANG--------------------------------- 384
Rel_Tn5386                    360 Q---TATVEDMIVKEKE------------------------- 373
Rel_ICECp1                    360 Q---TADTEELVTE---------------------------- 370
Rel_EfaC2                     414 V---SVDAKDLLFTNRD----NKSVRSYMEREFELEEMYPF- 447
Rel_ICE_SanC238_tRNAleu       394 ICERNGVDFDVIVGQI-------------------------- 409
Rel_ICE_Sdy12394_lysS         402 LKESYVPNHDEFVKGGKEWQVT-------------------- 423
Rel_ICE_SmiB6_guaA            385 Q---HLAMEDLIT----------------------------- 394
Rel_ICE_SparasFW213_ebfC      402 LKANKHYEH--------------------------------- 410
RepSTK1-4CIJ                  325 FKSKNITYDEMIKIIRQSK----------------------- 343
RepC_pT181                    303 IDFWQHEFKFWK------------------------------ 314
RepD_pC221                    300 IDFWQREFRFWK------------------------------ 311
RepN_pCW7                     303 IDFWQREFRFWK------------------------------ 314
Rep_pRS2_Ooeni                309 LKNWLQTFQVL------------------------------- 319
Rep_pK214_Llactis             268 IDFWHREFRFWE------------------------------ 279
Rep_pSP197_Staphpasteuri      358 TMIKAHEETKPFFK---------------------------- 371
Rep_pBt1-3_Bthuringiensis     338 FPIRDFKEFLKIYNEKCLK----------------------- 356
Rep_pMC5_Exiguobacterium      285 VVSWFKYAGVGVFQG--------------------------- 299
Rep_pMCCL4_Mcaseolyticus      292 LDFWLYEKETNFEKYFDFDTSEDLDQIQNRVIAKSKKVRGLE 333
Rep_pQY003_Emundtii           309 LDTWLQGLDVTEEGENNEL----------------------- 327
Rep_pJS42_Efaecium            302 LDTWLQGLDVTEVGAE-------------------------- 317
Rep_pF03-3_Lpentosus          282 LNGYLADFDIQK------------------------------ 293
```

**Figure S6. Sequence alignment of $MOB_T$ relaxases and *Rep_trans* RCR initiators.** The conserved motifs N', N", I, II, III and IV identified in Soler *et al*, 2019 (1) are boxed. Upper part: sequences of $MOB_T$ relaxases, bottom part: sequences of *Rep_trans* initiators. All sequences included in this alignment are detailed in Table S4. This sequence alignment was made using the Muscle program (2).

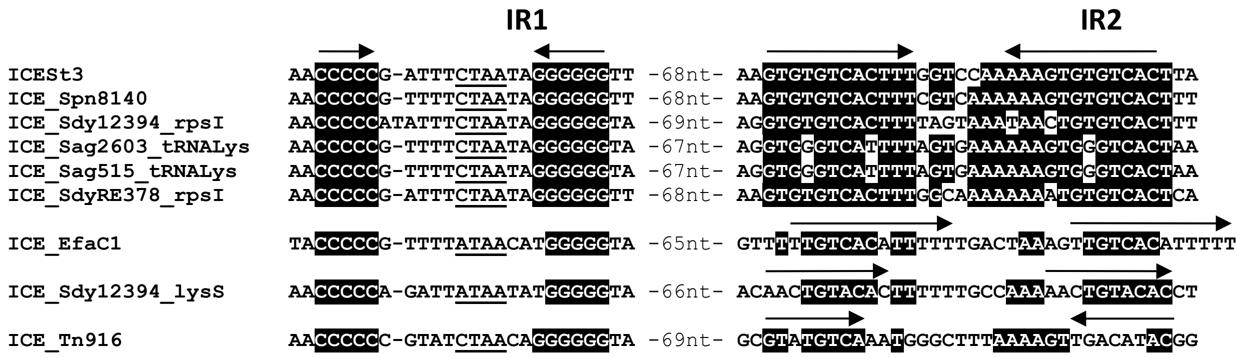


**Figure S9. Sequence conservation of putative IR2 *bind* sites in *oriT* sequences from different ICEs belonging to the ICE*St3* family and Tn*916*.** Most conserved positions are marked by white font on black background. Arrows indicate IR1 and IR2, or direct repeats (DR) in the case of the putative *bind* sites of ICE_EfaC1 and ICE_Sdy12394_lysS. The conserved nic site (C/ATAA) is underlined. The conserved distance between IR1 and IR2 is indicated for each ICE.

## Supplementary figure legends

**Figure S1. Estimation of RelSt3-DNA affinity constant.** Graphs reporting the quantification of DNA-replaxase complexes (in %) as a function of RelSt3 concentration were used to estimate the indicated apparent dissociation constants (Kd). (**A**) RelSt3 binding to ori41. (**B**) RelSt3 binding to ori50. Data were recovered from at least three experiments quantified with Image Lab software (BioRad), and the standard deviation is indicated. The affinity constant was estimated using the GraphPad software.

**Figure S2. Specificity of RelSt3 binding to ds-oriT41.** EMSAs reporting RelSt3 binding to ss-ori41 (**A**), ds-ori41 (**B**) or non-specific dsDNA (**C**). 5' 6-FAM labelled DNA (2pmol) was incubated with increasing concentrations of RelSt3: lane 1, 0 µM; lane 2, 0.1 µM; lane 3, 0.2 µM; lane 4, 0.4 µM; lane 5, 0.8 µM; lane 6, 1.6 µM; lane 7, 3.2 µM; lane 8, 6.4 µM.

**Figure S3. IR2 hairpin structure**. (**A**) IR2 structure prediction obtained using RNAfold software with DNA parameters ($\Delta G = -6.30\ kcal.mol^{-1}$. (**B**) Possible cruciform structure of IR2 dsDNA. (**C**) Nuclease S1 assays on ori41 dsDNA. Lane 1, 22 nt size marker; lane2 ori41; lanes 3 to 5, ori41 incubated with increasing amount of nuclease S1, respectively 0,05 U (lane 3), 0,5U (lane 4) and 2U (lane 5).

**Figure S4. Alphafold 3D model of RelSt3**. Front (upper panels) and side (bottom panels) views of the best-scored Alphafold model of Relt3. Left panels: 3D model colored according to the secondary structure elements (helices in pink and sheets in yellow). Middle panels: 3D model colored according to the per-residue confidence estimation of Alphafold (pLDDT: predicted Local Distance Difference Test), 0 = low confidence, 100 = high confidence. Right panels: 3D models colored according to the surface projection of electrostatic potential. Red, positively charged; white, uncharged; blue, negatively charged region.

**Figure S5. A complete *nic* site is needed for nicking-closing activities of RelSt3**. (**A**) Description of ori56 with respect to ori50 and ori57. Stars indicate the 6-FAM labelling positions. Unlabeled ori56 was annealed to unlabeled ori41c. (**B**) Nicking-closing activity assay with ori57 and ori56. Lane 1, 22 nt ss-DNA marker; lane 2,

59 nt ss-DNA ori57; lane3, ori50; lanes 4, ori50 and ori57 in the presence of RelSt3 (3.2µM) and $MnCl_2$ (positive control); lane 5, ori57 and ori56 in the presence of RelSt3 (3.2µM) and $MnCl_2$; lane 6, ori57 and ori56 without RelSt3 and $MnCl_2$.

**Figure S7. Secondary structure analysis of RelSt3 variants.** Far-UV spectra of RelSt3 WT and variants were recorded as described in Material and Methods, at 20°C from 190 to 260 nm (average of 3 scans) and converted to mean residue ellipticity.

**Figure S8. Formation of RelSt3-DNA covalent adduct is dependent on DNA concentration**. RelSt3 and unlabeled ori50 DNA were incubated as described in Materials and Methods in the presence of $Mn^{2+}$. M: Spectra multicolor broad range protein ladder (ThermoFischer). Lane 1: 5 µM of RelSt3 alone; lane 2: 5 µM of RelSt3 was incubated with 25 µM of ori50; lane 3: 5 µM of RelSt3 was incubated with 50 µM of ori50. The gel was stained with Coomassie blue. Red stars indicate the covalent RelSt3-DNA covalent complex.

**Figure S10. Alphafold 3D model of NicK from ICE*Bs1*.** Panels and colours are as described in Figure S4.

## Supplementary tables

### Table S1. Bacterial strains used in this work

| Strains | Genotype or description | Source or Reference |
|---|---|---|
| ***E. coli* strains** | | |
| EC101 | *supE hsd-5 thi* (*lac-proAB*) F (*traD6 proAB lacI*$^q$ *lacZ* M15) *repA*, derivative of strain TG1 (56) *repA*, derivative of strain JM101 | Laboratory stock, (3) |
| DH5α | $F^-$ Φ80*lac*ZΔM15 Δ(*lac*ZYA-*arg*F) U169 *rec*A1 *end*A1 *hsd*R17($r_k^-$, $m_k^+$) *pho*A *sup*E44 *thi*-1 *gyr*A96 *rel*A1 $\lambda^-$ | Invitrogen |
| BL21(DE3) | str. B $F^-$ *ompT gal dcm lon hsdS*$_B$($r_B^- m_B^-$) λ(DE3 [*lacI lacUV5-T7p07 ind1 sam7 nin5*]) [*malB*$^+$]$_{K-12}$($\lambda^S$) | Laboratory stock, (4) |
| ***S. thermophilus* strains** | | |
| LMG18311 (ICE*St3cat*) | LMG18311 strain carrying ICE*St3* tagged with the *cat* gene inserted in the pseudogene *Ψorf385J*, $Cm^r$ | (5) |
| LMG18311 (ICE*St3orfJΔHTH cat*) | LMG18311strain carrying ICE*St3* deleted for the N-terminal HTH domain (Δ4-189nt) in the *orfJ* gene, and tagged with the *cat* gene inserted in the pseudogene *Ψorf385J*, $Cm^r$ | This work |
| LMG18311 (pMG36e) | LMG18311 carrying pMG36e, a plasmid conferring erythromycin resistance | (5) |
| LMG18311 (ICESt3cat, pOri1180spec) | LMG18311 ICE*St3cat* strain carrying pOri1180spec conferring spectinomycin resistance | This work |

| | | |
|---|---|---|
| LMG18311 (ICESt3cat, pOri1180-*oriT1*) | LMG18311 ICE*St3cat* strain carrying pOri1180-*oriT1* conferring spectinomycin resistance and harboring the intergenic region *oriT1* between *orfK* and *orfJ* genes | (1) |
| LMG18311 (ICESt3cat, pOri1180-*oriT2*) | LMG18311 ICE*St3cat* strain carrying pOri1180-*oriT2* conferring spectinomycin resistance and harboring the *oriT2* sequence | This work |
| LMG18311 (ICESt3cat, pOri1180-*oriT3*) | LMG18311 ICE*St3cat* strain carrying pOri1180-*oriT3* conferring spectinomycin resistance and harboring the *oriT3* sequence | This work |
| LMG18311 (ICESt3cat, pOri1180-*oriT4*) | LMG18311 ICE*St3cat* strain carrying pOri1180-*oriT4* conferring spectinomycin resistance and harboring the *oriT4* sequence | This work |
| LMG18311 (ICESt3cat, pOri1180-*oriT5*) | LMG18311 ICE*St3cat* strain carrying pOri1180-*oriT5* conferring spectinomycin resistance and harboring the *oriT5* sequence | This work |

**Table S2. Plasmids used in this work**

| Plasmid | Description | Source or Reference |
|---|---|---|
| pTH24 | His-tagged-TEV protease coding sequence under control of T7/lac promoter | (6) |
| pSKB3 | Expression vector derived from pET28a including an in frame 6 His-tag and a TEV protease cleavage site in the N-terminal sequence | Gift from Stephen K. Burley |
| $pG^+Host9$ | 3.8 kb, pWV01-type thermosensitive replication origin from pVE6002, $Erm^r$ | (7) |
| $pG^+host9$*orfJ*$\Delta HTH$ | $pG^+Host9$ carrying the 1339 bp upstream region of *orfJ* CDS and 2060 bp, including from its $189^{th}$ nucleotide to its stop codon and the *orfJ* 1007 bp downstream region, $Erm^r$ | This work |
| pMG36e | 3.4 kb, replication origin from pWV01, $Ery^r$ | (8) |
| pOri1180 | pOri23 plasmid modified with a multiple cloning site from pSL1180-speclox conferring spectinomycin resistance | (9) and this work |
| pOri1180-*oriT1* | pOri1180 plasmid with ICE*St3* *oriT1* sequence cloned between *Eco*RI and *Apa*I restriction sites. | (1) |
| pOri1180-*oriT2* | pOri1180 plasmid with ICE*St3* *oriT2* sequence cloned between *Eco*RI and *Apa*I restriction sites. | This work |
| pOri1180-*oriT3* | pOri1180 plasmid with ICE*St3* *oriT3* sequence cloned between *Eco*RI and *Apa*I restriction sites. | This work |
| pOri1180-*oriT4* | pOri1180 plasmid with ICE*St3* *oriT4* sequence cloned between *Eco*RI and *Apa*I restriction sites. | This work |
| pOri1180-*oriT5* | pOri1180 plasmid with ICE*St3* *oriT5* sequence cloned between *Eco*RI and *Apa*I restriction sites. | This work |

## Table S3. Oligonucleotides used in this work

All oligonucleotides were purchased from Eurogentec, France. Restriction sites are underlined, and initiator codons are indicated with bold characters.

| Name | Sequence |
|---|---|
| **Oligonucleotides used for deletion of the N-terminal HTH domain in the *orfJ* gene in ICE*St3*** | |
| deltaRel_1 | GGGCGGCCGCAGGCTCTAAACTCCAACTTATG |
| deltaRelATG_2 | **CAT**TCATGGGAACACCTCCT |
| deltaHTHRel_3 | *AGGAGGTGTTCCCATGA**ATG***AAAAATAATCTTAGAGCACACATCGA<br>(the nucleotides overlapping with deltaRelATG_2 are represented in italics) |
| deltaRel_4 | CGGGGCCCACTTTATTTGAGAGCGTGATAG |
| K/J.For | TGTTCATCATGGGCTAGGAC |
| J/I.Rev | AAGGTACTTAGTCCACCTGACC |
| pGhost9For | CGCCATACCACAGATGTTCCAGATAAA |
| HindP | GACGTTGTAAAACGACGGCCAGT |
| **Oligonucleotides used for cloning of *orfJ* CDS segments into pSKB3 for over-expression of RelSt3$_{1-63}$ and RelSt3$_{64-410}$** | |
| St3-Rel-For | GGGAACATATGACTAAAATAAGTCCCTTTC |
| St3-RelHTH-Rev | ATCCTAAGCTTTTAATTATTTTTGTGACACTCTTGA |
| St3-ReldHTH-For | GGGAACATATGAAAAATAATCTTAGAGCACAAATC |
| St3-Rel-Rev | CGCAAAGCTTCTAGTGTTCATAGTGTTTGTTGG |
| **Oligonucleotide used for mutation of active site of RelSt3** | |
| ST3-Rel Y252A-Y256F For | GTCAGCTCTACTTCAACTTTGCTGAAAAGCGATTCGAAATCGCCCGTATGGAAAAT ATC |
| ST3-Rel Y252A-Y256F Rev | GATATTTTCCATACGGGCGATTTCGAATCGCTTTTCAGCAAAGTTGAAGTAGAGCT GAC |
| **Oligonucleotides used for cloning of ICE*St3 oriT1-oriT5* sequences in pOri1180 and generation of substrates for EMSA with agarose gels** | |
| St3-oriT1-For | AAAAAGAATTCTGTTCATCATGGGCTAGG |
| St3-oriT1-Rev | AAAAAGGGCCCTTGATCTGAAAGGGACTTATTT |
| St3-oriT2-For | AAAAAGAATTCCGGAGGAACAAGACCTCAA |
| St3-oriT2-Rev | AAAAAGGGCCCGAAATCGGGGGTTGAAGTC |
| St3-oriT3-For | AAAAAGAATTCTAATAGGGGGGTTACATTTGG |
| St3-oriT3-Rev | AAAAAGGGCCCCTTTGAGTTTTCGAAACTTGTC |
| St3-oriT4-For | AAAAAGAATTCAGGCATTGTATTCGAACGTTC |
| St3-oriT4-Rev | AAAAAGGGCCCGGAATGAGGAGGGGTGG |
| St3-oriT5-For | AAAAAGAATTCAATCCTCGCTCAATTTGA |
| St3-oriT5-Rev | AAAAAGGGCCCACCTCCTTTTGACTAAGTGACA |

**Oligonucleotide used in EMSA and activities tests**

| | |
|---|---|
| 22 nt marker | GAGACTTCAACCCCCGATTTCT |
| ori34 | TTTCAAGATTTAGAAAGTGTGTCACTTTGGTCCAAA |
| ori34C | TTTGGACCAAAGTGACACACTTTCTAAATCTTGAAA |
| ori43 | CTTTGGTCCAAAAAGTGTGTCACTTAGTCAAAAGGAG |
| ori43C | CTCCTTTTGACTAAGTGACACACTTTTTGGACCAAAG |
| ori46 | GAGACTTCAACCCCCGATTTCTAATAGGGGGGTTACAT |
| ori46C | ATGTAACCCCCCTATTAGAAATCGGGGGTTGAAGTCTC |
| ori47 | ACATTTGGCCAAAGTGCCACGTCCACCCCTCCTCATTCCTTGTGGGAGTTGGGATTTCAAGATTTAGA |
| ori47C | TCTAAATCTTGAAATCCCAACTCCCACAAGGAATGAGGAGGGGTGGACGTGGCACTTTGGCCAAATGT |
| ori41WT | TCAAGATTTAGAAAGTGTGTCACTTTGGTCCAAAAAGTGTGTCACTTAGTCAAAAGGAG |
| ori41C | CTCCTTTTGACTAAGTGACACACTTTTTGGACCAAAGTGACACACTTTCTAAATCTTGA |
| ori41M5 | TCAAGATTTAGACGATACGCTGACGTGGTCCAAAAAGTGTGTCACTTAGTCAAAAGGAG |
| ori41M5C | CTCCTTTTGACTAAGTGACACACTTTTTGGACCACGTCAGCGTATCGTCTAAATCTTGA |
| ori41M6 | TCAAGATTTAGAAAGTGTGTCACTTTGGTCCAAACGATACGCTGACGAGTCAAAAGGAG |
| ori41M6C | CTCCTTTTGACTCGTCAGCGTATCGTTTGGACCAAAGTGACACACTTTCTAAATCTTGA |
| ori41M11 | TCAAGATTTAGACGATACGCTATCGTGGTCCAAACGATACGCTATCGAGTCAAAAGGAG |
| ori41M11C | CTCCTTTTGACTCGATAGCGTATCGTTTGGACCACGATAGCGTATCGTCTAAATCTTGA |
| ori50 | GACTTCAACCCCCGATTTCTAATAGGGGGGTTACATTTTCAAGATTTAGAAAGTGTGTCACTTTGGTCCA AAAAGTGTGTCACTTAGTCAAAAGGAG |
| ori50M11 | GACTTCAACCCCCGATTTCTAATAGGGGGGTTACATTTTCAAGATTTAGACGATACGCTATCGTGGTCC AAACGATACGCTATCGAGTCAAAAGGAG |
| ori56 | AATAGGGGGGTTACATTTTCAAGATTTAGAAAGTGTGTCACTTTGGTCCAAAAAGTGTGTCACTTAGTC AAAAGGAG |
| ori57 | AGGCGCAGACCGTAGCCCGAAGTTCCTAGGCCATGATGAGACTTCAACCCCCGATTTCT |
| Non-specific DNA (Figure S2) | CTTACCGATAAGAACTTACCGCTCCTATGAATCTGGGGAAAGAGGTCTGACCATTGACAAGTTTCGAAA ACTCAAAGAAAAGC |
| Non-specific DNA C | GCTTTTCTTTGAGTTTTCGAAACTTGTCAATGGTCAGACCTCTTTCCCCAGATTCATAGGAGCGGTAAGT TCTTATCGGTAAG |

**Oligonucleotides used for DNA recombinant amplification and sequencing**

| | |
|---|---|
| Recomb-DNA For | CGCACATATGAGGCGCAGACCGTAGC |
| Recomb-DNA Rev | CGCAAAGCTTCTCCTTTTGACTAAGTGACACAC |

**Table S4. $MOB_T$ and *Rep_trans* sequences used for the alignment in Fig. S4.**

| Protein id | Name of ICE or plasmid | Organism |
|---|---|---|
| CAE52362 | ICE*St3* | *Streptococcus thermophilus* CNRZ385 |
| | ICE_*515_tRNA$^{Lys}$* | *Streptococcus agalactiae* 515 |
| AAB60013 | Tn*916* | *Enterococcus faecalis* |
| | ICE*Bs1* | *Bacillus subtilis* |
| ACF08822 | Tn*6009* | *Klebsiella pneumoniae* strain 41 |
| ACQ89867 | Tn*6202* | *Enterococcus faecalis* N00-410 |
| ACI48613 | ICE*6013* | *Staphylococcus aureus* ST239 |
| ADA65815 | Tn*6098* | *Lactococcus lactis* subsp. *lactis* KF147 |
| WP_015426015 | Nisin-sucrose transposon | *Lactococcus lactis* |
| NP_813946 | ICE*EfaC2* | *Enterococcus faecalis* V583 |
| CRG98343 | ICE*Cp1* | *Closridium perfringens* |
| AGU82651 | ICE_*SanC238_tRNA$^{leu}$* | *Streptococcus anginosus* C238 |
| ADX24159 | ICE_*Sdy12394_lysS* | *Streptococcus dysgalactiae* subsp. *equisimilis* ATCC 12394 |
| CBJ22567 | ICE_*SmiB6_guaA* | *Streptococcus mitis* B6 |
| AFJ26795 | ICE_*SparasFW213_ebfC* | *Streptococcus parasanguinis* FW213 |
| BAA06270 | pSTK1 | *Geobacillus stearothermophilus* |
| AAW36316 | pT181 | *Staphylococcus aureus* |
| CAA26104 | pC221 | *Staphylococcus aureus* |
| AAA26669 | pCW7 | *Staphylococcus aureus* |
| CAC67505 | pRS2 | *Oenococcus oeni* |
| CAA63520 | pK214 | *Lactococcus lactis* subsp. *lactis K214* |
| CAJ13696 | pSP197 | *Staphylococcus pasteuri* |
| AFV53176 | pBt1-3 | *Bacillus thuringiensis* serovar aizawai strain 1-3 |
| AHC04797 | pMC5 | *Exiguobacterium* sp. S3-2 |
| BAH18749 | pMCCL4 | *Macrococcus caseolyticus* JCSC5402 |
| BAO08778 | pQY003 | *Enterococcus mundtii* QU 25 |
| ABY76206 | pJS42 | *Enterococcus faecium* JH95 |
| AIG59283 | pF03-3 | *Lactobacillus pentosus* strain F03 |

## Supplementary References


1. Soler,N., Robert,E., Chauvot de Beauchêne,I., Monteiro,P., Libante,V., Maigret,B., Staub,J., Ritchie,D.W., Guédon,G., Payot,S., *et al.* (2019) Characterization of a relaxase belonging to the MOBT family, a widespread family in Firmicutes mediating the transfer of ICEs. *Mob. DNA*, **10**, 10.1186.

2. Edgar,R.C. (2004) MUSCLE: a multiple sequence alignment method with reduced time and space complexity. *BMC bioinformatics*, **5**, 113.

3. Leenhouts,K. (1995) Integration strategies and vectors. *Developments in biological standardization*, **85**, 523–530.

4. Studier,F.W. and Moffatt,B.A. (1986) Use of bacteriophage T7 RNA polymerase to direct selective high-level expression of cloned genes. *Journal of molecular biology*, **189**, 113–130.

5. Bellanger,X., Roberts,A.P., Morel,C., Choulet,F., Pavlovic,G., Mullany,P., Decaris,B. and Guédon,G. (2009) Conjugative transfer of the Integrative Conjugative Elements ICE*St1* and ICE*St3* from *Streptococcus thermophilus*. *Journal of Bacteriology*, **191**, 2764–2775.

6. van den Berg,S., Löfdahl,P.-Å., Härd,T. and Berglund,H. (2006) Improved solubility of TEV protease by directed evolution. *Journal of Biotechnology*, **121**, 291–298.

7. Maguin,E., Duwat,P., Hege,T., Ehrlich,D. and Gruss,A. (1992) New thermosensitive plasmid for gram-positive bacteria. *Journal of bacteriology*, **174**, 5633–5638.

8. van de Guchte,M., Van der Vossen,J.M., Kok,J. and Venema,G. (1989) Construction of a lactococcal expression vector: expression of hen egg white lysozyme in *Lactococcus lactis* subsp. *lactis*. *Applied and Environmental Microbiology*, **55**, 224–228.

9. Que,Y.-A., Haefliger,J.-A., Francioli,P. and Moreillon,P. (2000) Expression of *Staphylococcus aureus* clumping factor A in *Lactococcus lactis* subsp. *cremoris* using a new shuttle vector. *Infection and immunity*, **68**, 3516–3522.
